# Gegenees: Fragmented Alignment of Multiple Genomes for Determining Phylogenomic Distances and Genetic Signatures Unique for Specified Target Groups

**DOI:** 10.1371/journal.pone.0039107

**Published:** 2012-06-18

**Authors:** Joakim Ågren, Anders Sundström, Therese Håfström, Bo Segerman

**Affiliations:** 1 Department of Bacteriology, National Veterinary Institute (SVA), Uppsala, Sweden; 2 Department of Biomedical Sciences and Veterinary Public Health, Swedish University of Agricultural Sciences (SLU), Uppsala, Sweden; University of Hyderabad, India

## Abstract

The rapid development of Next Generation Sequencing technologies leads to the accumulation of huge amounts of sequencing data. The scientific community faces an enormous challenge in how to deal with this explosion. Here we present a software tool, ‘Gegenees’, that uses a fragmented alignment approach to facilitate the comparative analysis of hundreds of microbial genomes. The genomes are fragmented and compared, all against all, by a multithreaded BLAST control engine. Ready-made alignments can be complemented with new genomes without recalculating the existing data points. Gegenees gives a phylogenomic overview of the genomes and the alignment can then be mined for genomic regions with conservation patterns matching a defined target group and absent from a background group. The genomic regions are given biomarker scores forming a uniqueness signature that can be viewed and explored, graphically and in tabular form. A primer/probe alignment tool is also included for specificity verification of currently used or new primers. We exemplify the use of Gegenees on the *Bacillus cereus* group, on Foot and Mouth Disease Viruses, and on strains from the 2011 *Escherichia coli* O104:H4 outbreak. Gegenees contributes towards an increased capacity of fast and efficient data mining as more and more genomes become sequenced.

## Introduction

The scientific community faces an enormous challenge in how to efficiently exploit the huge amount of sequence data generated by the technological development of existing and new, next-generation sequencing (NGS) platforms. The rapid growth of NGS databases, in combination with more cost effective NGS instruments, gives intriguing opportunities both for research purposes and for clinical diagnostics. However, the demands on the genome analysis software are also increasing steeply. The algorithms and data storage must be highly efficient but it is also important to develop robust and intuitive user interfaces to manage and comprehend the massive datasets. NGS data analysis can give superior quality of comparison information spanning from distant phylogenetic relationships to the highest level of subtyping. From a comparative genomics perspective, a major challenge is to use very large datasets of NGS data to identify and visualize specific sequence signatures that represent scientifically or diagnostically relevant traits.

Producing a draft Whole Genome Shotgun (WGS) sequence assembly from a microbe has now become a standardized task and a similar development is anticipated for larger eukaryotic genomes. Draft WGS assemblies contain most of the genomic information although certain regions may be poorly covered. A common starting point when analyzing a new genome sequence is to place it in the phylogenetic/phylogenomic context of previously known genomes. It is often stated that phylogeny based on a larger number of genes/whole genome data is more reliable than phylogeny based on a single gene or a few selected loci [1]. At the same time, genomes often contain non-conserved genetic material that should be excluded to maximize the phylogenetic signal [2].

While a phylogenomic analysis can give an overall classification of a new genome sequence in the context of previous sequences, the next step of an analysis is usually aimed at obtaining detailed information on specific differences between genomes. Whole genome sequence alignment is not trivial and has consequently been an intensive research area the latest years [3], [4], [5], [6]. A commonly used program for pair-wise alignment of two genomes is MUMmer, which calculates Maximal Unique Matches, i.e., MUMs (which occur only once in both sequences). The requirements of uniqueness for the MUMs have been relaxed in more recent versions of MUMmer [7]. Other alignment programs often use the term MEMs for matches that occur one or more times in either sequence. MUMmer alignments can be visualized e.g., with dot plots. Multiple alignments of whole genome data usually involve identifications of ‘anchors’ that are series of substrings shared by the genomes to be aligned (e.g., MEMs). The alignment is then extended around the anchors using different alignment algorithms. One of the first softwares available for aligning three or more genomes was Multiple Genome Aligner (MGA) [8]. Since then, many programs with higher capacity and more efficient handling of draft sequences, rearrangements, duplications, and sequence gains and losses have been developed [9], [10]. Nonetheless, aligning large datasets is still challenging both in terms of required computational power and data interpretation.

When comparing multiple genomes, the problem can often be broken down to identifying and/or searching for a set of discriminating sequence motifs, i.e., a signature. The signature represents a single genome or a defined group of related genomes. From a clinical point of view this genome/group of genomes represents samples from the target group that we would like capture in a diagnostic assay (hereafter called the target group). Hence, we are looking for genomic signatures conserved in the target group but not present in other related or unrelated samples/genomes (hereafter called the background group). The presence of a target signature in a background sample/genome would represent a false positive and lack of conservation of the target signature would represent a false negative. The concept of defining target and background groups has been explored *e.g*., in the Insignia database using exact match alignments (MUMs/MEMs) produced by MUMmer [11]. Insignia contains most of the bacterial and viral genomes available from GenBank and pre-calculated exact sequence matches (of at least 18 bp in length) between the genomes. These MUMmer results are converted into a so-called “match cover” that is basically a list of intervals where there are continuous stretches of exact matches between genomes A and B. The user selects a group of target genomes of which one acts as reference genome. Instead of comparing all the whole genomes against each other at every query, Insignia compares the match covers to compose a list of signatures (MUMs/MEMs) that are shared by all target genomes and not found in the background genomes. These signatures can be filtered for several parameters including length and melting temperature and then used by the incorporated Primer3 software [12] for PCR-assay development. However, no unpublished genomes can be added and possibilities to explore longer and weaker conservation patterns are limited, since only perfectly matched regions are identified.

Here we present a new software package, Gegenees, that contributes towards solving some of the hurdles mentioned above. We have developed a stand-alone computer program with a graphical user interface (GUI) that covers the whole analysis chain, from obtaining and handling genome sequences, performing alignments, generating a phylogenomic overview, and visualizing and mining the alignment data in terms of target and background groups. Instead of using MUM/MEM anchors, we use a fragmented alignment procedure. The well-tried BLAST algorithm [13] is used for producing local alignments for up to billions of fragmented comparisons. Each data point connecting two genomes is represented by a score and not only by a present/absent relationship as in the MUM/MEM approach. To optimize speed, a multi-threaded blast control engine is used and the alignment is treated as a database that can be complemented with a new sequence without recalculating the entire alignment (as is usual for anchored alignments). The software is written in JAVA and is therefore compatible with several platforms (Linux, Macintosh and Windows). We also provide pre-calculated datasets that can be downloaded and complemented with custom data. The program was primarily developed for bacterial genomes but we have successfully aligned datasets with genomic sequences from virus, yeast, protozoa, and higher eukaryotic organisms.

## Methods

### Genome and Comparison Management

**Figure 1:**
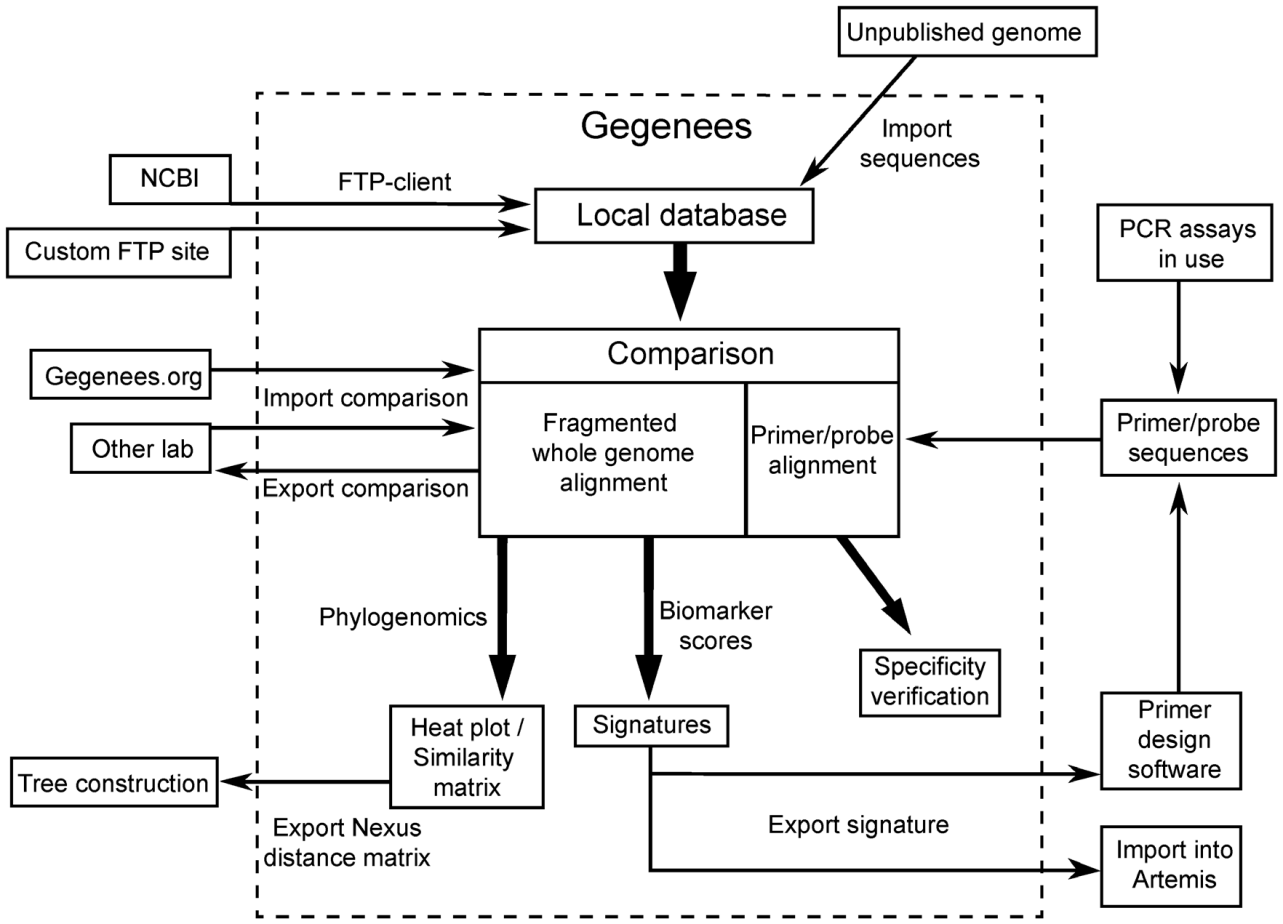
Overview of Gegenees. The Gegenees workspace contains one or several local databases. Genomes can be downloaded from the NCBI ftp site or from custom ftp sites through a built-in ftp client. This client compares the content of the local database with the remote one and highlights genomes already present locally. Unpublished genomes or genomes downloaded from other sources can be imported. The Gegenees workspace can also contain comparison projects. Genomes are added to the comparison from the local database. Genomes already in the active comparison are highlighted in the local database to facilitate the update process. Comparisons can also be downloaded from www.gegenees.org or shared between labs and imported into the workspace. One or several fragmented alignments can be made in the comparison with custom-specified resolution. Large alignments are associated with lengthy calculations and can therefore be paused and later resumed. Genomes can also later be added to a completed alignment that is then updated with the missing data points. When an alignment has been completed, the phylogenomic context can be analyzed in heat-plots. Nexus files can be exported for dendrogram construction and heat plots can be exported for high-resolution printouts. The alignment can then be analyzed in terms of Biomarker scores and uniqueness signatures. A target and a background group are defined on the basis of strain phenotypes and phylogenomic overview. The resulting conservation pattern signature can then be viewed and explored graphically or in tables. The signatures can also be exported to Artemis. Primers and/or probes can be designed from the signatures and candidate primers can be added back to Gegenees in form of a primer/probe alignment. Primer specificity can then be analyzed in terms of mismatches in the target and background groups.

Gegenees not only addresses the hurdle of aligning hundreds of genomes. The software has also been designed to streamline the task of managing and keeping a collection of perhaps thousands of genomes up-to-date, as well as a large number of comparison projects. The software uses a graphical user interface and is compatible with Linux, Macintosh and Windows. An overview of the functionality of Gegenees is shown in Figure 1. The latest version of Gegenees can be downloaded at www.gegenees.org.

Completed and draft bacterial genomes are collected in different directories at the National Center for Biotechnology Information (NCBI) ftp server. The constant addition of new genomes and the inconsistent and often cryptic file-naming conventions make it a tedious task to manually keep track of changes. The Gegenees ftp client can be used to conveniently download genomes from the NCBI ftp site. Cryptic genome names like wgs.ADCZ.1.gbff.gz are converted to the actual name of the species and strain. Genomes already present in the local database are marked red in the remote listing. This enables efficient comparison between local and remote data so that the local data can be kept up to date. The ftp or database lists can contain thousands of genomes and therefore possibilities to filter the views have been implemented. Gegenees can also be configured to use local ftp servers as long as they follow the NCBI ftp-site format. The genomes are stored in one or several local databases. Gegenees also includes import functions for handling unpublished genomic data. In summary, Gegenees addresses the question of how to efficiently manage and keep up-to-date datasets as well as perform comparisons of ever-growing numbers of genomes.

### A Multi-threaded Control Engine Managing Fragmented BLAST Alignments of Hundreds of Genomes

Gegenees does not use an anchored alignment process. Rather, a fragmented approach is used to make all-against-all whole genome comparisons. This kind of fragmented comparison approach (e.g., sliding-window based) has previously been used to compare pair-wise average similarities of genome sequences [14]. In Gegenees, all genomes are converted into datasets of fragments that together represent all the genomic regions from each genome in the comparison. The resolution of the alignment is controlled by two parameters: the fragment-size (i.e., the sliding-window size) and the step-size. The best BLAST [13] alignment for each fragment against every genome in the comparison (one by one) is then calculated and collected into a database. The power of the approach lies in the amount of data-points. An alignment of 150 genomes with an average size of 5 Mbp using a 100 bp step-size gives over one billion data-points. Both BLASTN (nucleotide comparison) and TBLASTX (translated comparison) can be used for alignments.

A set of small and overlapping fragments gives superior resolution when searching for short genome signatures but the calculation becomes more demanding. In a bacterial genome, the average length of a gene is typically between 900–1000 bp. To obtain several fragments from most genes, we use a fragment length of 200 and a step-size of 100 as standard values (hereafter referred to as ‘200/100’) but we also find it valuable to use non-overlapping 500 bp fragments (hereafter referred to as ‘500/500’) for faster, but less accurate, alignments. Using a too small window size can yield a low signal-to-noise ratio because the max-score of the alignment becomes closer to the background score [14]. This elevated background is mainly seen with the older BLAST (blastall) implementation. The newer BLAST+ [15] gives less increase in the background scores when using short fragments. We routinely use a 50 bp fragment-size and a 25 bp step-size for alignment of viral genomes without problems. If the only purpose is to get a fast phylogenomic overview of a group of genomes, the step-size may be set larger than the fragment-size. This will yield a phylogeny based on a sampling of the genomes which can give sufficient resolution but will preclude meaningful signature analysis.

Fragmented alignments are sequentially performed in Gegenees, analyzing one fragment at a time, and therefore do not use up as much of the computer’s RAM memory as anchored, whole-genome alignment programs often do. In principle, given enough time and disk space, any size of alignment could be made. A lengthy alignment can also be paused, and continued later, if the computer is needed for other purposes and it can be resumed after a crash. An expert user can also relatively easily divide a large calculation onto several computers. A major advantage is also that new genomes can be easily added to an existing alignment by only complementing the missing data-points. To simplify the process for end users with limited computational capacity, ready-made genome comparisons for the larger bacterial genera have been placed at the Gegenees remote resource (www.gegenees.org).

**Figure 2:**
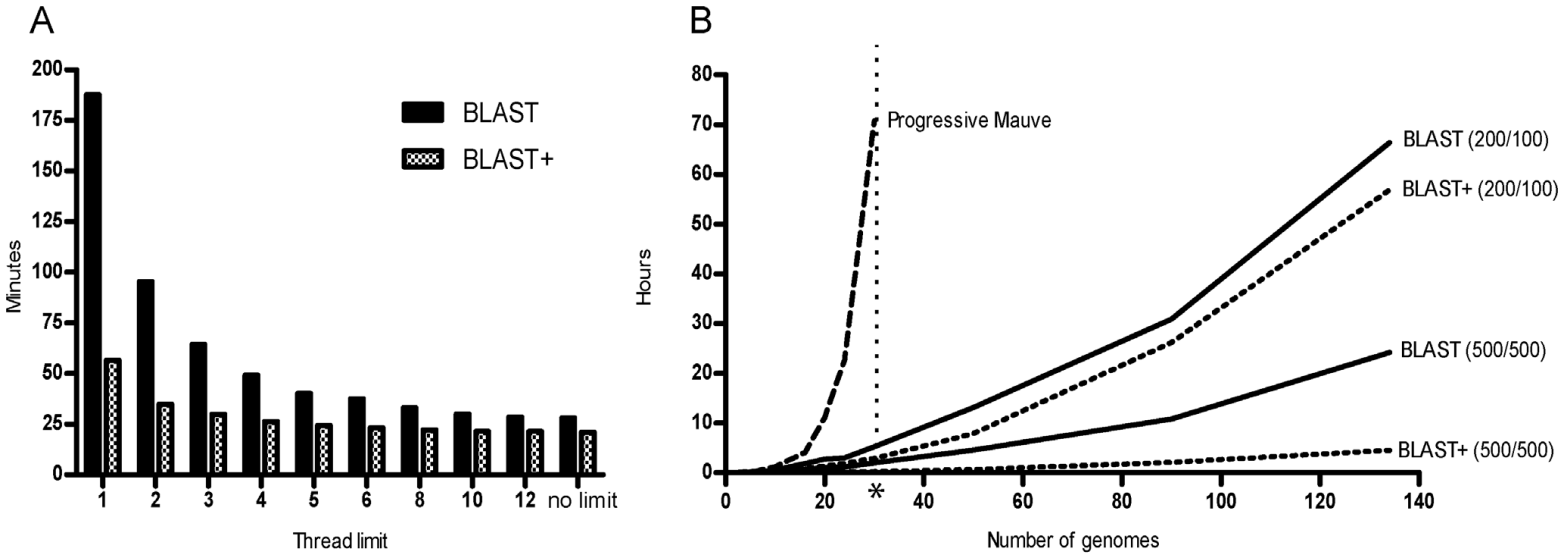
Gegenees calculation speed. Calculation benchmark made on a workstation equipped with a 3.2 GHz Intel i7 -970 processor (6 cores with hyper-threading, i.e., 12 simultaneous threads). **A.** The Gegenees source code was modified so that the number of simultaneous threads was limited to 1, 2, 3…. The time for completing a comparison with 10 *Bacillus*.spp genomes (∼5 Mb each) with BLAST (blastall) or BLAST+ was measured. When no thread limit was used Gegenees chose to use 12 threads on this machine. **B**. Time required for completing an alignment with an increasing number of *Bacillus* spp. genomes. Progressive Mauve (version 2.3.1) with default settings and Gegenees with different settings (500/500 or 200/100 using blastall or BLAST+) were compared. The asterisk indicates the upper limit of genomes (30) we could align in Progressive Mauve on this machine.

To maximize calculation speed, the BLAST control engine in Gegenees has been made multi-threaded which means that the program sends a new BLAST job to each CPU core as soon as one is available. Doing so makes full use of the modern multi-core CPUs. This can reduce the calculation time to 15–37% percent of the single-threaded time or more, depending on BLAST version and processor architecture. In Figure 2A the times for aligning ten *Bacillus* genomes (∼5 Mbp genomes, 200/100 setting) are plotted with different ‘maximum number of threads’ limits and this shows that calculation time is drastically reduced when using multiple parallel threads. Especially the older BLAST (blastall) implementation is accelerated several-fold, but BLAST+ [15] calculation time is also reduced to less than half by Gegenees multi-threading capacity. In our hands, the acceleration that the Gegenees multi-threading gives cannot be replaced by modifying the ‘-a’ or ‘num_threads’ options in BLAST. BLAST+ is a much faster implementation compared to BLAST, especially when using longer fragments, and it is therefore highly recommended to use the BLAST+ implementation with Gegenees.

### Phylogenomic Overview of Data and Management of Target/Background Groups

To efficiently formulate target groups that can be used to identify genomic regions with important conservation patterns, it is valuable to get an overview of the overall relationship between the genomes in an alignment. By summarizing all pair-wise average similarities in the alignment into a matrix, such an overview can be obtained. When one genome is compared to another, the average score of all fragments, or alternatively from all fragments over a certain threshold, is used as a measurement of similarity. The data are normalized against the maximum score that can be obtained with the fragment. The threshold is used to obtain a stronger phylogenomic signal since it removes non-conserved regions from the analysis. Thresholds can be set to 5, 10, 15, 20, 25, 30, 35 or 40% of the maximum score value. Also, if no threshold is used, comparing two genomes of different size can sometimes give large differences in values depending on whether the small one is compared to the large one or the other way around.

In Gegenees, the similarity matrix is displayed as a heat-plot. The color profile and the number of decimals can be adjusted so that optimal views of datasets with different properties can be obtained. The size of the “core genome” at a certain threshold can also be viewed as a heat plot. Data can be exported as a plain table, as an HTML heat plot table, or in the Nexus file format for dendrogram production. The HTML heat-plot format can easily be converted to publication-grade quality images.

An important function of the heat plot is to give the user an overview of average similarities. As the datasets get larger, functions for sorting and comparing phylogeny with the definition of the target and background groups become more and more important. Gegenees has therefore been equipped with tools for manual and automated sorting; the composition of the target and the background groups can be directly viewed and modified from the heat-plot.

### Mining an Alignment for Genomic Regions Characteristic or Unique for a Specified Target Group

A Gegenees alignment can be mined for genomic regions that are characteristic or unique for a specified target group. The target group, consisting of one or more genomes, is defined in a table and/or in the heat-plot view. The groups are defined on the basis of knowledge of the strain phenotypes and the phylogenomic overview. To connect the description of a target signature to a coordinate system and to give annotations, a reference genome must be defined within the target group. The choice of reference genome does not, in most cases, affect the number of unique genomic fragments found for a target group since the regions found by Gegenees are shared by all target genomes. However, if the target group conservation constraint is relaxed, the choice of reference genome can have a certain importance.

The basis for the signature analysis is to compare the conservation pattern between the target and background groups for every fragment from the currently selected reference genome. All fragments are given a biomarker score that can be plotted along the reference genome coordinate system and/or sorted in tabular form. In the stringent form of the biomarker score, a fragment’s maximum score against the background group (worst false positive) is divided by the minimum BLAST score against the target group genomes (worst false negative) and finally subtracted from 1 to yield a result ranging from 1 (perfect conservation and no cross-reaction) to −∞. For practical purposes, scores below 0 (a worst cross-reaction in the background is a better target than the least-conserved target group genome) are considered bad, and Gegenees only plots the range between 0 and 1 in the graphical overview. In some cases the high stringency biomarker score (max/min) is too stringent, *e.g.* if the dataset contains poor quality sequences, if uncertainties in the target group definition exist, or if there are no fully unique genomic regions present. Therefore, Gegenees provides two less stringent scores, namely (max/average) and (average/average). If no notable biomarker score profile is visible with the stringent biomarker score, it can be valuable to switch to these less stringent forms. The (max/average) biomarker score is based on average values in the target group instead of minimum values and will therefore include regions in the signature which may be absent or less conserved in a portion of the target group. It is then possible to define the less conserved genome(s) through the “detailed score” function in the tabular view. If the less conserved genome is a draft, it is possible that the signature may be located in a sequence gap in that assembly and therefore still be fully valid. In the least stringent form of biomarker score (average/average), the average score of the background group is used instead of the maximum. A few of the background genomes are then allowed to contain the signature and a certain level of cross reactivity is tolerated (false positives). Still there may exist sub-regions within the signature that can be highly specific. The background group and target group conservations can also be viewed independently. Genomes can also be excluded from the biomarker analysis if they do not fit in either the target or the background group.

In Gegenees, the biomarker scores can be plotted along the coordinate system of the reference genome. To enable direct comparison of two biomarker stringencies or background and target group conservations independently, graphs can be plotted both above and below the coordinate-axis. An analysis is typically started with an overview of the entire reference genome. Each pixel-column in the diagram might contain several fragments and if so, the maximum score or the average score of those fragments can be viewed. A zoom function enables detailed analysis of specific regions. Regions can be selected with the mouse for further examination or manipulation in the table view. The biomarker scores can also be exported in a format with color coded features that can be viewed in Artemis [16]. An example is shown in Figure S1. This allows detailed comparison between the biomarker signature and the annotation features.

The table view enables detailed analysis of the fragment properties, positions, annotations, scorings, etc. The content of the table can be filtered using biomarker score thresholds and/or specific fragment ranges. The nucleotide sequence of selected fragments can be viewed, exported, or sent to the NCBI BLAST web-server. Fragments can be viewed independently or processed so that overlapping and adjacent fragments are fused. The exported sequences can be used to design primers and probes for the development of new diagnostic assays. This design is done in the preferred primer-design software outside Gegenees. A list of candidate primers and probes can then be put back into Gegenees in the form of a “primer alignment”. The primer alignment aligns all candidate primers against all genomes in the comparison using BLASTN with a short sequence setting. The results are summarized in a table where the “non-alignment index”, meaning the sum of non-aligned nucleotides and reported mismatches, is shown for each genome in the comparison. The target group and the background group can be color coded so that the specificity profile can be determined. Identity, query length, alignment length, and mismatches are also shown and the actual alignment can be viewed so that the relative positions of mismatches can be analyzed.

For more in-depth details regarding how Gegenees works, see Text S1.

### Sequences

All sequences used in this study were downloaded from GenBank. Accession numbers of the genomes used in this study are given in Tables S1,S2,S3 and S5,S6,S7.

## Results

### Gegenees Comparison Performance

To put the efficiency of Gegenees in relation to other whole-genome alignment software, an increasing number of *Bacillus* spp. genomes were analyzed both with Progressive Mauve [10] and Gegenees (with both the 500/500 and 200/100 settings). The computer had a six-core Intel i7 CPU, 12 GB of RAM and running SUSE Linux operating system, i.e., represented a fast desktop workstation. The total analysis time and memory usage were recorded for each run. The results in Figure 2B show that Gegenees fragmented alignment is superior to Progressive Mauve (anchored alignment) in terms of calculation speed and limitations on the number of genomes. The amount of memory used by Gegenees for the comparison of four genomes was the same as for 134 genomes. On the same computer, we could not align more than 30 genomes in Progressive Mauve due to memory requirement errors. The alignment-size independent memory usage and the multi-threading acceleration ensure a high scalability and enable Gegenees to compare hundreds of genomes on a normal workstation computer. (We have without problems compared all available 189 *Streptococcus* genomes on a standard workstation in 37 hours with 200/100 settings). However, since Gegenees do not analyze rearrangement patterns, the analysis is advantageously followed up or complemented by anchored alignments. An example of an analysis using Gegenees, Mauve and Mugsy [9] on a dataset of Helicobacter pylori genomes is described in Text S2.

### Gegenees Phylogenomic Overview Compared to Previous Studies

**Figure 3:**
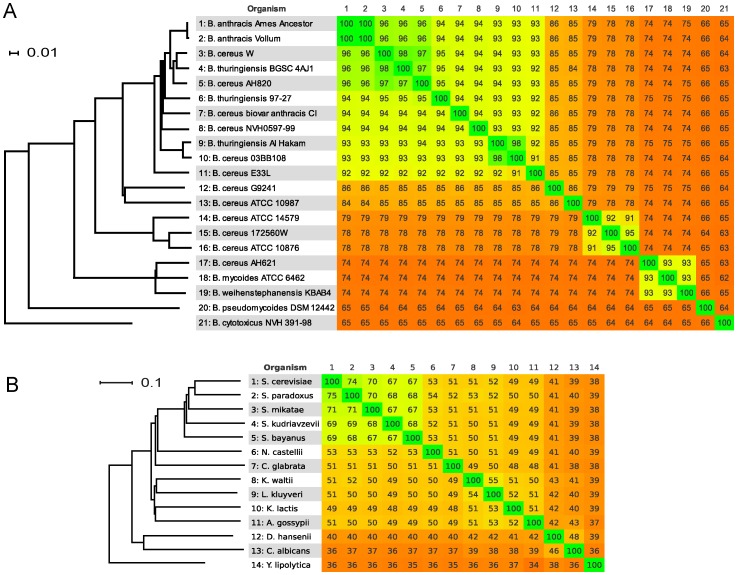
Phylogenomic overview in Gegenees. Both heat-plots of the similarity matrices and trees created from the same data are shown. **A**. A Gegenees heat-plot over a set of *Bacillus* strains that had previously been analyzed by MLST [17]. The heat-plot is based on a fragmented alignment using BLASTN made with settings 200/100. The cutoff threshold for non-conserved material was 30%. A dendrogram was produced in SplitsTree 4 (using neighbor joining method) made from a Nexus file exported from Gegenees. *B. cytotoxicus* was set as outgroup. The clustering is very similar to previously published trees. The scale bar represents a 1% difference in average BLASTN score similarity. **B**. A Gegenees heat-plot over a set of yeast genomes that has been analyzed before with different phylogenomic methods. These genomes are more distant from each other and a BLASTN comparison does not resolve them well (data not shown). A fragmented alignment in TBLASTX mode was performed with settings 200/200. The cutoff threshold for non-conserved material was 20%. A dendrogram was produced in SplitsTree 4 (using neighbor joining method) made from a distance matrix Nexus file exported from Gegenees. *Y. lipolytica* was set as outgroup. The clustering here is also very similar to the previously published trees [2]. The scale bar represents a 10% difference in average TBLASTX score similarity.

The resolving power of a nucleotide comparison is good for strains within the same species and between closely related ones. In Figure 3A, a BLASTN comparison of a set of related *Bacillus* spp. strains (Table S1) that had previously been analyzed using MLST [17], is shown. To more comprehensively compare the tree derived from Gegenees-data with a tree created using MLST, the concatenated sequences of 7 housekeeping genes from these 21 strains were downloaded from the Bacillus cereus group MLST database for the Tourasse-Helgason scheme [18], and a tree was created in MEGA [19] using Maximum-Likelihood. The MLST tree showed the same clustering of strains as the tree created from Gegenees-data (Figure S5 and Figure 3A). The clustering of *Bacillus*-strains based on the *panC* gene sequences, proposed by Guinebretiere et al [20] is also supported in both the Gegenees and the MLST based trees.

More distantly related genomes can be analyzed using a TBLASTX alignment. In Figure 3B, 14 yeast genome sequences (Table S2) that previously had been put in a phylogenetic context by Jeffroy et al. [2], were analyzed using Gegenees in TBLASTX mode. In conclusion, the Gegenees similarity matrices gave phylogenetic clustering very similar to those produced by other established methods.

### Alignment and Analysis of 134 *Bacillus* Genomes with Gegenees

One of the largest genera represented in the bacterial genome databases at the time of this analysis was *Bacillus* (134 genomes). We included an analysis of the *Bacillus* genus because of the large number of genomes available and because it is a phylogenetically very complex group. For a complete list of the genomes included in this *Bacillus-*dataset, and their status of completion, see Table S3.

*Bacillus* is a genus with gram-positive, rod-shaped bacteria commonly found in the environment. *B. anthracis* (the causative agent of anthrax) and *B. cereus* (which causes food-borne illness) are medically important species. They are both members of the group *Bacillus cereus*-*sensu lato* (*B. cereus, B. anthracis, B. thuringiensis, B. mycoides*, *B. weihanstephanensis* and *B. pseudomycoides*) which has a very intermixed phylogenetic structure. The validity of these species definitions has been debated frequently [21], [22]. *B. anthracis* is a Biosafety Level 3 (BSL3) organism that constitutes an example of a challenging target group in terms of biomarker identification since the *B. anthracis* strains are very closely related to other *B. cereus sensu lato* strains in the background group [22], [23], [24], [25]. Because of this and given that both *B. anthracis* and closely related strains are well represented in the genome database, we chose this species for detailed study.

**Figure 4:**
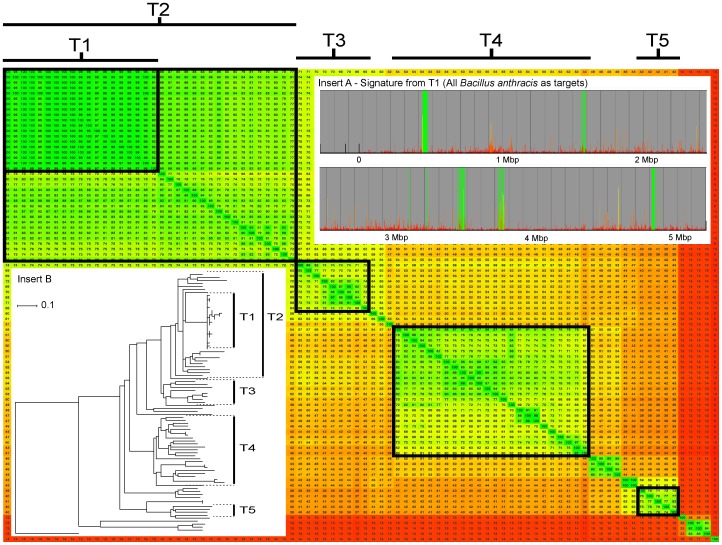
Comparative analysis of the *Bacillus cereus* group. A heat-plot based on a 200/100 BLASTN fragmented alignment without threshold is shown. The figure is cropped to show only the *Bacillus cereus* group. Target groups used for PCR design are indicated (T1–T5). All remaining *Bacillus* genomes were used as a background group. This analysis was made without a threshold to filter non-conserved genetic material. Viewing the heat-plot without a threshold means that the values are based on both the core genome size and the core conservation. This often gives a better view during target group formulation because signatures are per definition outside the core when comparing a target genome with a background genome. Insert A shows the uniqueness signature for *B. anthracis* (T1). Signatures for all groups are present in Figure S3. Insert B shows a dendrogram based on the heat plot. The dendrogram was produced in SplitsTree 4 (using neighbor joining method) made from a distance matrix Nexus file exported from Gegenees. *B. cytotoxicus* was set as outgroup.

A fragmented genome alignment of the 134 *Bacillus*-genomes was performed with a window-size of 200 bp and a step-size of 100 bp. This produced around 50,000 fragments for each strain. The full phylogenomic heat-plot overview created from the alignments can be seen in the supporting information (Figure S2). To find unique signatures for anthrax, we chose the 14 available *B. anthracis* genomes as a target group and all other *Bacillus* spp. as a background group. The amount of unique genomic material was, as expected, low. No high biomarker scores were found in the plasmids since some of the sequenced *B. anthracis* strains lacked one or both of the virulence plasmids. If the target group was reformulated so that all strains with a specific plasmid were set as target, biomarker scores could be obtained for the plasmids as well (data not shown). There were five larger and a few much shorter chromosomal regions with high biomarker scores when using this target group (Figure 4, insert A). By setting a biomarker score threshold at 0.8, we found 555 fragments (corresponding to about 1% of the fragments) unique to *B. anthracis*. Four of the regions were related to the prophages Ba01, Ba02, Ba03 and Ba04 that have been described in the literature as unique for *B. anthracis* [26], [27]. The Ba03 prophage has been used to design a highly specific chromosomal *B. anthracis* PCR assay [28]. The fifth large region of high conservation amongst the *B. anthracis* strains is a stretch of roughly 28 kb that contains genes coding for a flagellar-capping protein, a flagellin, and a glycosyl transferase. An export to Artemis of this region is shown in Figure S1.

To validate the primer design approach of Gegenees in practice, five distinct, but closely related target groups (T1, T2, T3, T4 and T5), were chosen within the *Bacillus cereus sensu lato* group (Figure 4 and Table S3). For each target group, three independent real-time PCR assays were designed targeting different genomic-signature regions. All five signatures are shown in Figure S3. All primer candidates were used in a setup of real-time PCR using a template panel of 17 different *Bacillus* species that had been genome sequenced (Table S4). A total of 255 PCRs were performed. Fifty-four of them were expected to give positive results. In practice, it turned out that 51 reactions gave a positive signal (Table S4). The primers were also analyzed using the Gegenees primer alignment function. All three failures could unequivocally be predicted from the primer alignment analysis, since there were one or several base mismatches in the 3′ end of the primer. Thus, we could conclude that the primer design approach of Gegenees is clearly applicable for this kind of assay development.

The primer sequences and PCR-conditions can be seen in the supplemental material and methods (Text S1).

### Analysis of an Alignment of 254 Foot-and-Mouth Disease Viruses (FMDV)

We also wanted to test the performance of Gegenees on viral genomes. FMDV is an ssRNA-virus from the *Picornaviridae* family, genus *Aphthovirus*, which is responsible for the extremely contagious animal disease, Foot-and-Mouth disease. There are seven identified serotypes: O, A, C, Asia1, and the South African Territories (SAT) 1, SAT2 and SAT3. These serological differences can be attributed the four capsid proteins VP1–VP4 of which VP1 is the dominating attributor. VP1-sequences have also been used for genotyping [29].

**Figure 5:**
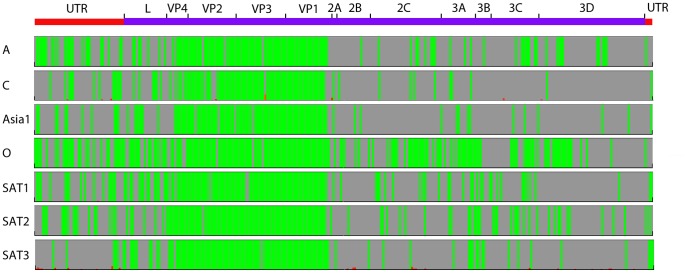
Signature analysis of Foot-and-Mouth Disease Virus (FMDV) serotypes. A fragmented alignment was performed with 50/25 settings using BLASTN (BLAST+). Target groups were formulated according to the serotype definitions. All other serotypes were used as background. The ‘maximum background/average target’ setting was used for biomarker score calculation. The annotations shown come from the type Asia 1 isolate IND 13–91(DQ989312). VP1–VP4 constitutes the capsid proteins that are exposed on the virus particle and are therefore important determinants for serotype classification.

We used Gegenees to align 254 FMDV genomes (Table S5) with typical sizes of approximately 8,200 bp. For viruses, the signatures are expected to be smaller. We therefore used a window-size of 50 bp and a step-size of 25 bp. The alignment was performed in approximately 1 hour on a standard workstation. We then wanted to search the genomes for signatures specific for the seven serotypes. We therefore defined seven target groups corresponding to the serotypes. A stringent biomarker signature could not be defined, but when using the max/average biomarker score, we could identify regions with higher specificity for the different serotypes (Figure 5). The majority of the signature signal was located in the VP1–VP4 region, which makes sense since it is the region containing the capsid proteins. The heat-plot showed clustering that broadly followed the serotypes but some exceptions were also found indicating recombination sometimes occurs between serotypes (data not shown) which has been observed before [30].

### Analysis of the *E. coli* O104:H4 Genome from the 2011-outbreak in Europe

A major advantage of Gegenees is that a new genome sequence can be added into a pre-calculated alignment. This means that a new genome can be compared to a very large number of genomes with only modest calculation time, which is important from a preparedness perspective. As new genome sequences become available, they can be continually checked against a large reference data set, for instance to ensure the continued specificity of diagnostic markers. We used the 2011 European outbreak of highly virulent *Escherichia coli* O104:H4 that caused serious complications such as hemolytic-uremic syndrome (HUS) to exemplify the advantage of pre-calculated data. Starting with a pre-calculated dataset of 95 *Escherichia* genomes (available from NCBI, but not including the outbreak strain), we added the draft sequence of the outbreak isolate LB226692 (NCBI accession AFOB02000000), to the pre-calculated Gegenees comparison in 40 minutes on a standard workstation. The accession numbers from the genomes are listed in Table S6.

The phylogenomic overview is shown in Figure S4. The outbreak isolate LB226692 shows a close relationship with the enteroaggregative *E. coli* (EAEC) 55989 strain (NCBI accession NC_011748) and the historical O104 HUS isolate 01-09591. This is in concordance with a previous phylogenetic analysis [31].

**Figure 6:**
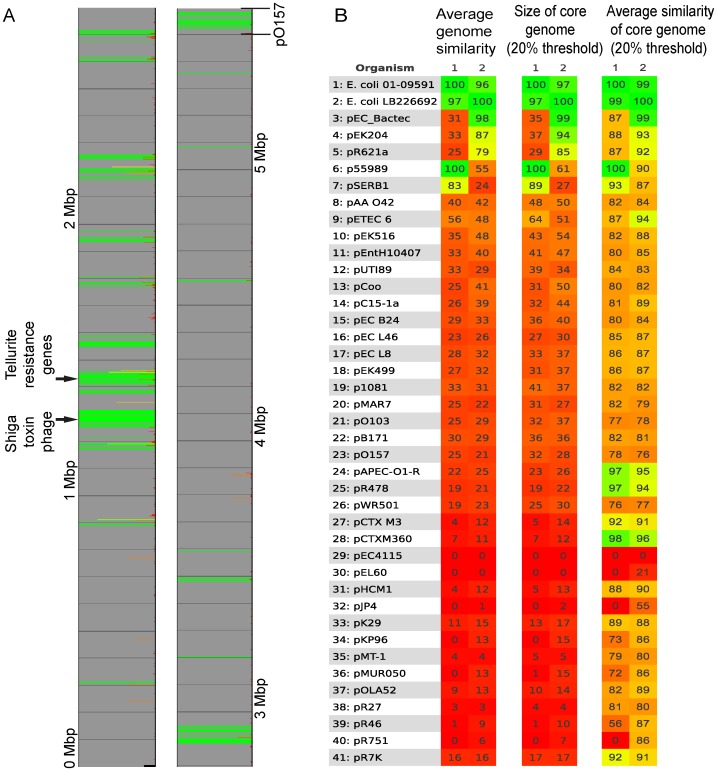
Signature analysis of the *E. coli* O104:H4 strain from the food poisoning outbreak in 2011. A. A signature representing the genetic material that the outbreak strain LB226692 (accession AFOB02000000) has in common with previous severe food-poisoning outbreak strains (Sakai Japan 1996 (accession NC_002695), Michigan and Oregon 1982 (accession NC_002655), the spinach outbreak in western USA 2006 (accession NC_013008) and the lettuce outbreak in eastern USA 2006 (accession NZ_ABKY00000000)) but not in common with a background strain representing another *E. coli* O104 strain 55989 (accession NC_011748). **B.** Plasmid profiling using Gegenees. Two O:104 isolates, one from the 2011 outbreak (LB226692) and the other a HUS-associated O104 strain from 2001 (accession AFPS01000000), were compared to a set of plasmids with a fragmented alignment 200/100 using BLASTN.

To illustrate how Gegenees can be used in comparative genomics on a draft assembly, a signature was produced that showed the genetic material that the outbreak isolate shared with pathogenic O157:H7 isolates from previous serious outbreaks in humans [32], [33], but at the same time not shared with the EAEC 55989 strain (Figure 6A). This signature included the Shiga toxin-producing phage and the tellurite resistance-coding genes (*ter*). Thus, this type of analysis can quickly give insights in what the outbreak isolate has acquired from other groups of pathogenic *E. coli*.

Plasmids play an important role in transferring virulence factors and we wanted to illustrate how Gegenees can be used for plasmid typing. A dataset of 39 known *E. coli* related plasmids (Table S7) was used, to which the 2011 O104 outbreak isolate LB226692 and the 2001 O104 HUS isolate 01-09591 were added for the plasmid comparison. The first two columns from heat-plots of the total average similarity, the average similarity of the conserved core (threshold 20%) and the size of the core (threshold 20%) are shown in Figure 6B. From the heat-plot it is possible to see that the LB226692 isolate had acquired a plasmid highly similar to the entire pEC_Bactec plasmid. The reason for the high scores for plasmids pEK204 and pR621a is because they are very similar to the pEC_Bactec [34]. This could be recognized as such from the cluster between these three plasmids in the full heat-plot (data not shown). The 01-09591 isolate contained plasmids similar to the entire p55989 plasmid and 90% of the pSERB1 plasmid (Figure 6B). This is in concordance with previous reports [31]. In conclusion, a database of all plasmids relevant for *E. coli* can be complemented with the assembly of a sequenced outbreak strain. Plasmid (or chromosomal plasmid material) content within the outbreak strain can then be identified in minutes.

## Discussion

An anchored alignment gives information on conservation pattern and rearrangements but is limited in the number of genomes that can be analyzed. In the fragmented alignment, the coordinate information is only saved for the query. The hit coordinates are discarded and information about rearrangements is therefore not available. Rearrangements are frequent in bacterial genomes and there is a constant reshuffling of genes symmetrically around the origin of replication [35]. This can be seen as a cross-pattern in a dot plot of a pair-wise alignment. In most cases, the gene content, rather than the gene locations seems to be most important for phenotype. Frequent rearrangements can also obscure the conservation pattern analysis of large numbers of genomes in traditional alignments. However, if the rearrangement patterns are important, anchored alignment analysis should be used. It is our belief that a Gegenees analysis can advantageously be followed up by complementary anchored alignments, once the overall picture is defined.

In a sense, a fragmented alignment is similar to a gene-content analysis but it takes into account all DNA in the genome and it is also possible to find sub-regions in the conserved genes. The fragmented alignment approach is also less sensitive to frame-shifts compared to analysis of gene predictions, which makes draft genome analysis more robust. In conclusion we believe that Gegenees can fill an important function during the upcoming years as the inflow of new genome sequences increases. We believe that a precalculated dataset of reference strains with predefined target groups can quickly answer clinically important questions in terms of the presence or absence of virulence associated signatures. Molecular probes and primers in clinical use can also easily be checked against a constantly growing database in terms of specificity. This version of Gegenees is mostly developed for microorganisms, but we believe that there is a potential to use the same approach for analysis of higher eukaryotes as well.

## Supporting Information

Figure S1 **A genomic signature for** ***B. anthracis*** **exported from Gegenees and imported in Artemis.** One of the few *B. anthracis*-specific genomic regions is shown.
(PDF)

Figure S2 **Average whole genome similarity of the** ***Bacillus*** **-genus.** A heat-plot showing the similarity matrix when comparing 134 *Bacillus* spp. genomes with Gegenees.
(PDF)

Figure S3 **Genomic target areas for PCR-design.** The genomic signatures, as shown by Gegenees, for the five target groups (T1–T5) in the *Bacillus* genus. The target groups are listed in Table S3.
(PDF)

Figure S4 **Average whole genome similarity of the** ***Escherichia*** **-genus.** A heat-plot showing the similarity matrix when comparing 97 *Escherichia* spp. genomes with Gegenees.
(PDF)

Figure S5 ***Bacillus cereus*** **group MLST tree.** Maximum-Likelihood tree created in MEGA 5.05 using the Tamura-Nei nucleotide substitution model. The 7 housekeeping-gene sequences used for each strain were from the Tourasse-Helgason MLST scheme. The tree shows the clustering of 21 whole genome sequenced Bacillus cereus-group members that were also used to create Figure 3A. The roman numerals in the parentheses indicates the Bacillus-clustering based on the panC gene sequences proposed by Guinebretiere et al. and the following number indicates the ST given for the strain by the MLST database (http://mlstoslo.uio.no). Scale bar represents nucleotide substitutions per site.
(PDF)

Table S1 **A list of** ***Bacillus*** **spp. genomes and their accession numbers, used in** **Figure 3A** **.**
(PDF)

Table S2 **A list of yeast genomes and their accession numbers, used in** **Figure 3B** **.**
(PDF)

Table S3 ***Bacillus*** **spp. genomes used in the** ***Bacillus*** **comparison.** Target groups (T1–T5) are indicated.
(PDF)

Table S4 **Templates and PCR results from the** ***Bacillus*** **spp. target groups T1**–**T5.** The target groups are listed in Table S3. T1, T2, T3, T4 and T5 represent the target groups used to produce signatures and PCR-assays from. S1, S2 and S3 represent the three PCR-primer-pairs chosen from each of the five signatures. Field highlighted in green represents PCR-positive reaction, as expected from PCR-design. Field highlighted in orange represents PCR-negative reaction, as expected from PCR design. Field highlighted in red represents PCR-negative reaction, in contradiction to the positive results expected from PCR-design.
(PDF)

Table S5 **A list of Foot and Mouth disease Virus (FMDV) genomes used in the serotype comparisons.**
(PDF)

Table S6 **A list of** ***Escherichia*** **spp. genomes used in the** ***Escherichia*** **comparison.**
(PDF)

Table S7 **A list of** ***Escherichia*** **strains and plasmids used in the** ***Escherichia*** **plasmid comparison.**
(PDF)

Text S1 **In-depth material and methods.** Text describing the structure of files used/created by Gegenees, the workflow and settings of the BLAST runs and also the PCR setup.
(PDF)

Text S2 **Comparison between Gegenees, Mauve and Mugsy.** Text describing an analysis example with Gegenees, Mauve and Mugsy. The highly plastic genomes of the available Helicobacter pylori strains were used to show how fragmented and anchored alignments performs and complements each other when used with highly rearranged sequences.
(PDF)
