# Supplementary figures and images for: Gegenees: Fragmented Alignment of Multiple Genomes for Determining Phylogenomic Distances and Genetic Signatures Unique for Specified Target Groups
Source: PLoS One. 2012 Jun 18;7(6):e39107. doi: 10.1371/journal.pone.0039107 (PMC3377601; doi:10.1371/journal.pone.0039107)

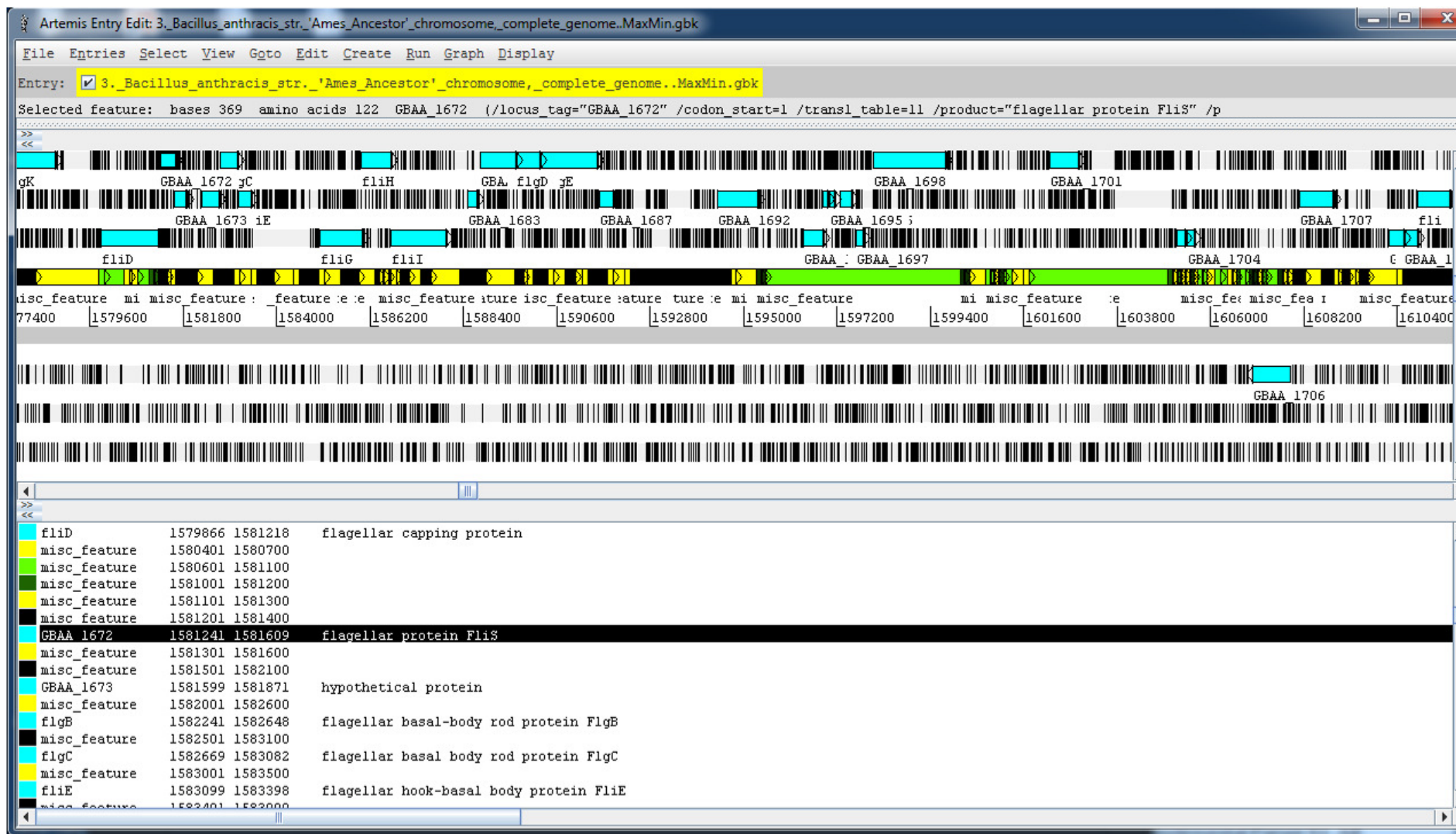

Supplement: Figure S1 — A genomic signature for B. anthracis exported from Gegenees and imported in Artemis. One of the few B. anthracis-specific genomic regions is shown. (PDF) [file pone.0039107.s001.pdf]

0.01

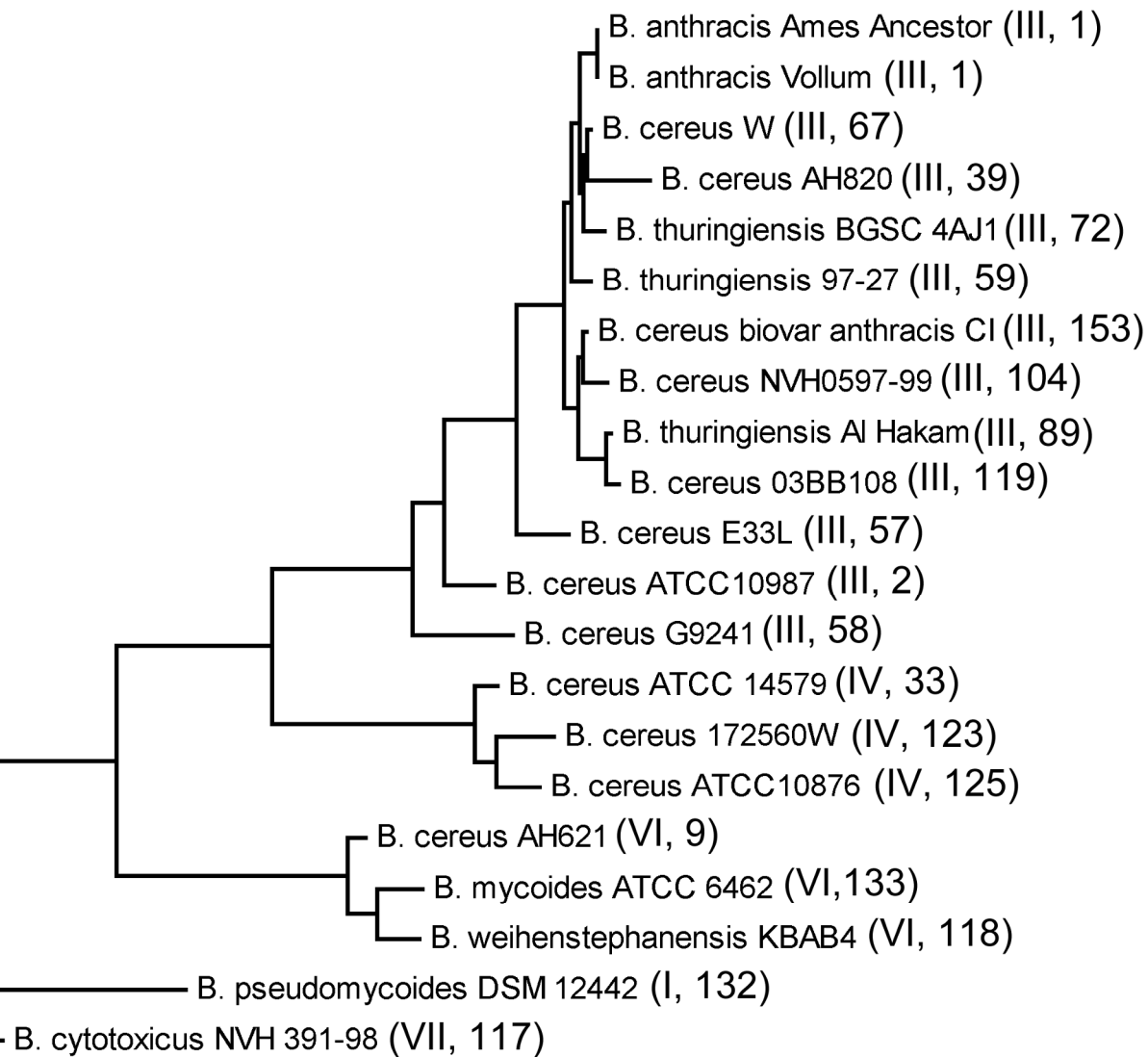

Supplement: Figure S5 — Bacillus cereus group MLST tree. Maximum-Likelihood tree created in MEGA 5.05 using the Tamura-Nei nucleotide substitution model. The 7 housekeeping-gene sequences used for each strain were from the Tourasse-Helgason MLST scheme. The tree shows the clustering of 21 whole genome sequenced Bacillus cereus-group members that were also used to create Figure 3A. The roman numerals in the parentheses indicates the Bacillus-clustering based on the panC gene sequences proposed by Guinebretiere et al. and the following number indicates the ST given for the strain by the MLST database (http://mlstoslo.uio.no). Scale bar represents nucleotide substitutions per site. (PDF) [file pone.0039107.s008.pdf]
