# Supplementary material for: Gegenees: Fragmented Alignment of Multiple Genomes for Determining Phylogenomic Distances and Genetic Signatures Unique for Specified Target Groups
Source: PLoS One. 2012 Jun 18;7(6):e39107. doi: 10.1371/journal.pone.0039107 (PMC3377601; doi:10.1371/journal.pone.0039107)
Supplement: Figure S3 — Genomic target areas for PCR-design. The genomic signatures, as shown by Gegenees, for the five target groups (T1–T5) in the Bacillus genus. The target groups are listed in Table S3. (PDF) [file pone.0039107.s005.pdf]

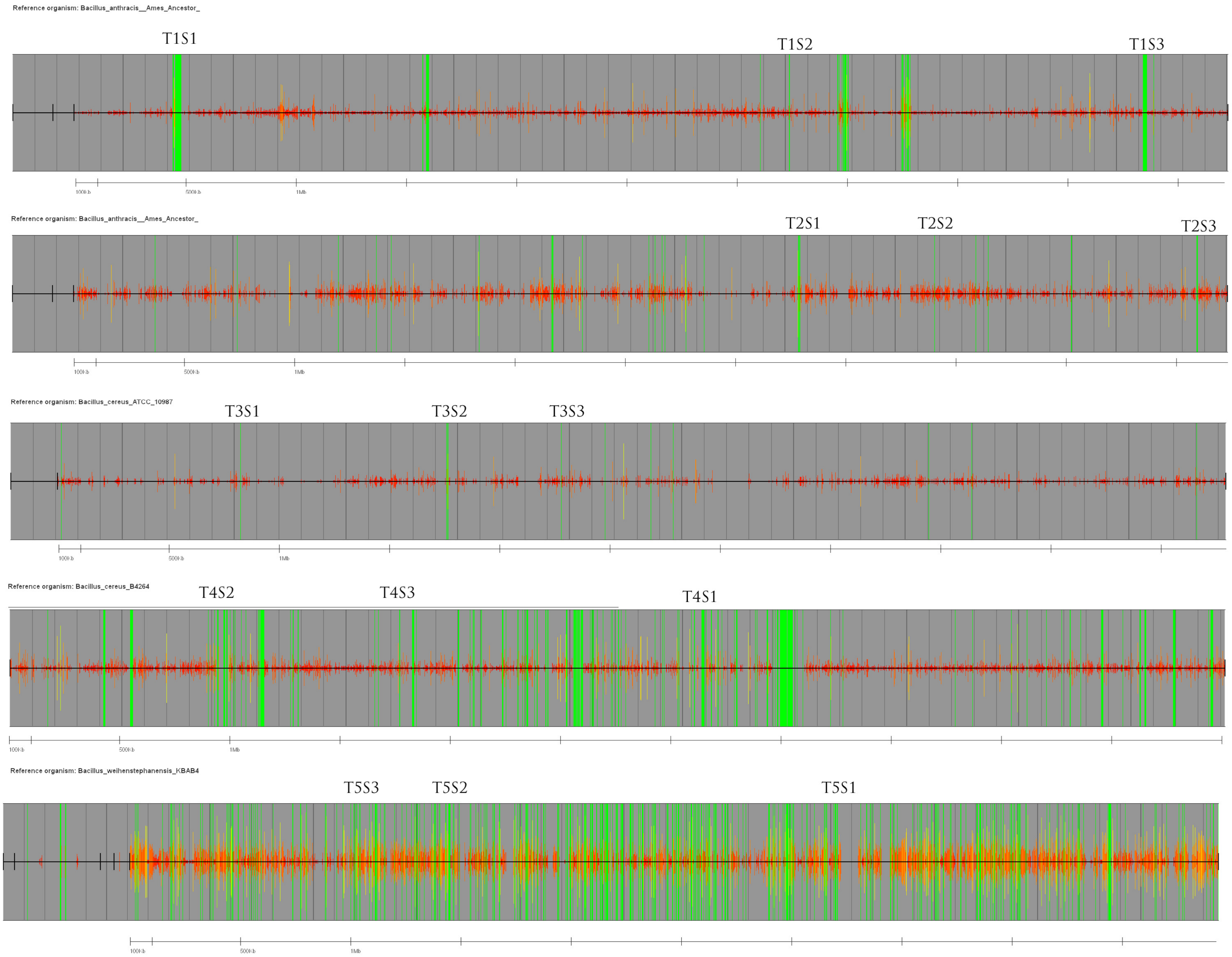

Reference organism: Bacillus_anthracis__Ames_Ancestor_
T1S1
T1S2
T1S3
100Kb
500Kb
1Mb
Reference organism: Bacillus_anthracis__Ames_Ancestor_
T2S1
T2S2
T2S3
100Kb
500Kb
1Mb
Reference organism: Bacillus_cereus_ATCC_10987
T3S1
T3S2
T3S3
100Kb
500Kb
1Mb
Reference organism: Bacillus_cereus_B4264
T4S2
T4S3
T4S1
100Kb
500Kb
1Mb
Reference organism: Bacillus_weihenstephanensis_KBAB4
T5S3
T5S2
T5S1
100Kb
500Kb
1Mb
