# Supplementary material for: Gegenees: Fragmented Alignment of Multiple Genomes for Determining Phylogenomic Distances and Genetic Signatures Unique for Specified Target Groups
Source: PLoS One. 2012 Jun 18;7(6):e39107. doi: 10.1371/journal.pone.0039107 (PMC3377601; doi:10.1371/journal.pone.0039107)
Supplement: Table S1 — A list of Bacillus spp. genomes and their accession numbers, used in Figure 3A . (PDF) [file pone.0039107.s010.pdf]

Supplemental Table S1
A list of *Bacillus* Spp. genomes and their accession numbers used in Figure 3A

Genome	State	No. of. subsequences/contigs	NCBI accession number
Bacillus cereus W	Draft	102	NZ_ABCZ
Bacillus weihenstephanensis KBAB4	Complete	5	NC_010183, NC_010180, NC_010182, NC_010181, NC_010184
Bacillus cereus ATCC 10987	Complete	2	NC_005707, NC_003909
Bacillus cereus ATCC 14579	Complete	2	NC_004721, NC_004722
Bacillus anthracis Ames Ancestor	Complete	3	NC_007322, NC_007323, NC_007530
Bacillus cereus AH820	Complete	4	NC_011776, NC_011771, NC_011777, NC_011773
Bacillus thuringiensis serovar monterrey BGSC 4AJ1	Draft	250	NZ_ACNE
Bacillus cereus ATCC 10876	Draft	245	NZ_ACLT
Bacillus cereus 03BB108	Draft	66	NZ_ABDM
Bacillus cereus NVH0597 99	Draft	71	NZ_ABDK
Bacillus anthracis Vollum	Draft	52	NZ_AAEP
Bacillus thuringiensis serovar konkukian 97 27	Complete	2	NC_005957, NC_006578
Bacillus thuringiensis Al Hakam	Complete	2	NC_008598, NC_008600
Bacillus cereus G9241	Draft	207	NZ_AAEK
Bacillus pseudomycoides DSM 12442	Draft	305	NZ_ACMX
Bacillus cytotoxicus NVH 391 98	Complete	2	NC_009674, NC_009673
Bacillus cereus 172560W	Draft	152	NZ_ACLV
Bacillus cereus AH621	Draft	190	NZ_ACLX
Bacillus cereus biovar anthracis CI	Complete	4	NC_014331, NC_014332, NC_014335, NC_014333
Bacillus cereus E33L	Complete	6	NC_007103, NC_007106, NC_007104, NC_007105, NC_007107, NC_006274
Bacillus mycoides ATCC 6462	Draft	200	NZ_ACMU
