# Supplementary material for: Gegenees: Fragmented Alignment of Multiple Genomes for Determining Phylogenomic Distances and Genetic Signatures Unique for Specified Target Groups
Source: PLoS One. 2012 Jun 18;7(6):e39107. doi: 10.1371/journal.pone.0039107 (PMC3377601; doi:10.1371/journal.pone.0039107)
Supplement: Table S2 — A list of yeast genomes and their accession numbers, used in Figure 3B . (PDF) [file pone.0039107.s011.pdf]

Supplemental Table S2

A list of yeast genomes and their accession numbers used in Figure 3B

Genome	State	No. of. subsequences/contigs	NCBI accession number
Ashbya gossypii ATCC 10895	Complete	8	NC_005782, NC_005783, NC_005784, NC_005785, NC_005786, NC_005787, NC_005788, NC_005789
Candida glabrata	Complete	14	NC_004691, NC_005967, NC_005968, NC_006026, NC_006027, NC_006028, NC_006029, NC_006030, NC_006031, NC_006032, NC_006033, NC_006034, NC_006035, NC_006036
Candida albicans SC5314	Draft	413	AACQ
Debaryomyces hansenii CBS767	Complete	8	NC_006043, NC_006044, NC_006045, NC_006046, NC_006047, NC_006048, NC_006049, NC_010166
Kluyveromyces lactis NRRL Y-1140	Complete	7	NC_006037, NC_006038, NC_006039, NC_006040, NC_006041, NC_006042, NC_006077
Kluyveromyces waltii NCYC 2644	Draft	713	AADM
Lachancea kluyveri NRRL Y 12651	Draft	36	AACE
Naumovozyma castellii CBS 4309	Complete	10	NC_016491, NC_016500, NC_016492, NC_016493, NC_016494, NC_016495, NC_016496, NC_016497, NC_016498, NC_016499
Saccharomyces cerevisiae	Complete	17	NC_001133, NC_001134, NC_001135, NC_001136, NC_001137, NC_001138, NC_001139, NC_001140, NC_001141, NC_001142, NC_001143, NC_001144, NC_001145, NC_001146, NC_001147, NC_001148, NC_001224
Saccharomyces bayanus MCYC 623	Draft	1098	AACA
Saccharomyces kudriavzevii IFO 1802	Draft	2029	AACI
Saccharomyces mikatae IFO 1815	Draft	1648	AABZ
Saccharomyces paradoxus NRRL Y 17217	Draft	832	AABY
Yarrowia lipolytica CLIB122	Complete	7	NC_002659, NC_006067, NC_006068, NC_006069, NC_006070, NC_006071, NC_006072
