# Supplementary material for: Gegenees: Fragmented Alignment of Multiple Genomes for Determining Phylogenomic Distances and Genetic Signatures Unique for Specified Target Groups
Source: PLoS One. 2012 Jun 18;7(6):e39107. doi: 10.1371/journal.pone.0039107 (PMC3377601; doi:10.1371/journal.pone.0039107)
Supplement: Table S3 — Bacillus spp. genomes used in the Bacillus comparison. Target groups (T1–T5) are indicated. (PDF) [file pone.0039107.s012.pdf]

Supplemental Table S3

A list of *Bacillus* Spp. genomes used in the *Bacillus* comparison. Target groups (T1-T5) are also indicated.

Target group for PCR 1-5, 0 = background	Genome	State	No. of. subsequences/contigs	NCBI accession number
1,2	Bacillus anthracis A1055	Draft	42	NZ_AAEO
1,2	Bacillus anthracis CDC 684	Complete	3	NC_012579, NC_012581, NC_012577
1,2	Bacillus anthracis CNEVA 9066	Draft	30	NZ_AAEN
1,2	Bacillus anthracis A0174	Draft	60	NZ_ABLT
1,2	Bacillus anthracis A0442	Draft	46	NZ_ABKG
1,2	Bacillus anthracis Ames Ancestor	Complete	3	NC_007322, NC_007323, NC_007530
1,2	Bacillus anthracis Australia 94	Draft	49	NZ_AAES
1,2	Bacillus anthracis A0389	Draft	68	NZ_ABLB
1,2	Bacillus anthracis Kruger B	Draft	64	NZ_AAEQ
1,2	Bacillus anthracis Tsiankovskii I	Draft	62	NZ_ABDN
1,2	Bacillus anthracis Sterne	Complete	1	NC_005945
1,2	Bacillus anthracis Ames	Complete	1	NC_003997
1,2	Bacillus anthracis Vollum	Draft	52	NZ_AAEP
1,2	Bacillus anthracis A0488	Draft	63	NZ_ABJC
1,2	Bacillus anthracis A0193	Draft	60	NZ_ABKF
1,2	Bacillus anthracis A0465	Draft	57	NZ_ABLH
1,2	Bacillus anthracis A0248	Complete	3	NC_012655, NC_012659, NC_012656
1,2	Bacillus anthracis A2012	Draft	1	NZ_AAAC
1,2	Bacillus anthracis Western North America USA6153	Draft	44	NZ_AAER
2	Bacillus cereus 03BB102	Complete	2	NC_012472, NC_012473
2	Bacillus cereus 03BB108	Draft	66	NZ_ABDM
2	Bacillus cereus 95 8201	Draft	228	NZ_ACMF
2	Bacillus cereus AH820	Complete	4	NC_011776, NC_011771, NC_011777, NC_011773
2	Bacillus cereus BGSC 6E1	Draft	202	NZ_ACLU
2	Bacillus cereus biovar anthracis CI	Complete	4	NC_014331, NC_014332, NC_014335, NC_014333
2	Bacillus cereus E33L	Complete	6	NC_007103, NC_007106, NC_007104, NC_007105, NC_007107, NC_006274
2	Bacillus cereus NVH0597 99	Draft	71	NZ_ABDK
2	Bacillus cereus SJ1	Draft	254	NZ_ADFM
2	Bacillus cereus W	Draft	102	NZ_ABCZ
2	Bacillus thuringiensis Al Hakam	Complete	2	NC_008598, NC_008600
2	Bacillus thuringiensis serovar andalousiensis BGSC 4AW1	Draft	257	NZ_ACNG
2	Bacillus thuringiensis serovar konkukian 97 27	Complete	2	NC_005957, NC_006578
2	Bacillus thuringiensis serovar monterrey BGSC 4AJ1	Draft	250	NZ_ACNE
2	Bacillus thuringiensis serovar pondicheriensis BGSC 4BA1	Draft	185	NZ_ACNH
2	Bacillus thuringiensis serovar pulsiensis BGSC 4CC1	Draft	258	NZ_ACNJ
2	Bacillus cereus Rock3 42	Draft	192	NZ_ACMK
3	Bacillus cereus ATCC 4342	Draft	105	NZ_ACLZ
3	Bacillus thuringiensis serovar tochigiensis BGSC 4Y1	Draft	219	NZ_ACMY
3	Bacillus cereus AH187	Complete	5	NC_011654, NC_011658, NC_011657, NC_011656, NC_011655
3	Bacillus cereus ATCC 10987	Complete	2	NC_005707, NC_003909
3	Bacillus cereus G9241	Draft	207	NZ_AAEK
3	Bacillus cereus Q1	Complete	3	NC_011971, NC_011969, NC_011973
3	Bacillus cereus BDRD ST26	Draft	182	NZ_ACMC
3	Bacillus cereus H3081 97	Draft	80	NZ_ABDL
3	Bacillus cereus m1293	Draft	241	NZ_ACLS
4	Bacillus cereus 172560W	Draft	152	NZ_ACLV
4	Bacillus cereus AH676	Draft	315	NZ_ACMQ
4	Bacillus cereus ATCC 10876	Draft	245	NZ_ACLT
4	Bacillus cereus ATCC 14579	Complete	2	NC_004721, NC_004722
4	Bacillus cereus B4264	Complete	1	NC_011725
4	Bacillus cereus BDRD Cer4	Draft	117	NZ_ACME
4	Bacillus cereus BDRD ST24	Draft	185	NZ_ACMB
4	Bacillus cereus F65185	Draft	267	NZ_ACMO
4	Bacillus thuringiensis BMB171	Complete	2	NC_014171, NC_014172
4	Bacillus thuringiensis Bt407	Draft	187	NZ_ACMZ
4	Bacillus thuringiensis IBL 200	Draft	244	NZ_ACNK
4	Bacillus thuringiensis IBL 4222	Draft	383	NZ_ACNL
4	Bacillus thuringiensis serovar berliner ATCC 10792	Draft	253	NZ_ACNF
4	Bacillus thuringiensis serovar huazhongensis BGSC 4BD1	Draft	278	NZ_ACNI
4	Bacillus thuringiensis serovar israelensis ATCC 35646	Draft	866	NZ_AAJM
4	Bacillus thuringiensis serovar kurstaki T03a001	Draft	337	NZ_ACND
4	Bacillus thuringiensis serovar pakistani T13001	Draft	617	NZ_ACNC
4	Bacillus thuringiensis serovar sotto T04001	Draft	481	NZ_ACNB
4	Bacillus thuringiensis serovar T01001	Draft	246	NZ_ACNA
4	Bacillus cereus AH1134	Draft	37	NZ_ABDA
4	Bacillus cereus G9842	Complete	3	NC_011775, NC_011774, NC_011772

4	Bacillus cereus m1550	Draft	124	NZ_ACMA
4	Bacillus cereus Rock1 15	Draft	199	NZ_ACMH
4	Bacillus cereus Rock4 2	Draft	323	NZ_ACMM
5	Bacillus cereus AH603	Draft	251	NZ_ACMP
5	Bacillus cereus AH621	Draft	190	NZ_ACLX
5	Bacillus mycoides DSM 2048	Draft	200	NZ_ACMU
5	Bacillus weihenstephanensis KBAB4	Complete	5	NC_010183, NC_010180, NC_010182, NC_010181, NC_010184
5	Bacillus cereus BDRD ST196	Draft	284	NZ_ACMD
0	Bacillus 2 A 57 CT2	Draft	114	NZ_ACWD
0	Bacillus cereus MM3	Draft	197	NZ_ACLW
0	Bacillus amyloliquefaciens DSM 7	Complete	1	NC_014551
0	Bacillus amyloliquefaciens FZB42	Complete	1	NC_009725
0	Bacillus atrophaeus 1013-1	Draft	28	AEFS
0	Bacillus atrophaeus 1013-2	Draft	28	AEFT
0	Bacillus atrophaeus 1942	Complete	1	NC_014639
0	Bacillus atrophaeus ATCC 49822-1	Draft	31	AEFV
0	Bacillus atrophaeus ATCC 49822-2	Draft	30	AEFW
0	Bacillus atrophaeus ATCC 9372-1	Draft	28	AEFM
0	Bacillus atrophaeus ATCC 9372-2	Draft	32	AEFU
0	Bacillus atrophaeus BACI051-E	Draft	35	AEFX
0	Bacillus atrophaeus BACI051-N	Draft	78	AEFY
0	Bacillus atrophaeus Detrick-1	Draft	31	AEFP
0	Bacillus atrophaeus Detrick-2	Draft	47	AEFQ
0	Bacillus atrophaeus Detrick-3	Draft	30	AEFR
0	Bacillus atrophaeus str. Dugway	Draft	53	AEFO
0	Bacillus B14905	Draft	99	NZ_AAXV
0	Bacillus BT1B CT2	Draft	49	NZ_ACWC
0	Bacillus cellulosilyticus DSM 2522	Complete	1	NC_014829
0	Bacillus cereus AH1272	Draft	416	NZ_ACMS
0	Bacillus cereus AH1273	Draft	412	NZ_ACMT
0	Bacillus clausii KSM K16	Complete	1	NC_006582
0	Bacillus coagulans 2 6	Complete	1	NC_015634
0	Bacillus coagulans 36D1	Complete	1	NC_016023
0	Bacillus coagulans XZL4	Draft	232	AFWM
0	Bacillus coahuilensis m4 4	Draft	138	NZ_ABFU
0	Bacillus cytotoxicus NVH 391 98	Complete	2	NC_009674, NC_009673
0	Bacillus halodurans C 125	Complete	1	NC_002570
0	Bacillus licheniformis ATCC 14580	Complete	1	NC_006270
0	Bacillus m3 13	Draft	50	NZ_ACPC
0	Bacillus megaterium DSM319	Complete	1	NC_014103
0	Bacillus megaterium QM B1551	Complete	8	NC_010009, NC_014023, NC_014025, NC_010008, NC_010010, NC_014031, NC_004604, NC_014019
0	Bacillus NRRL B 14911	Draft	109	NZ_AAOX
0	Bacillus pumilus ATCC 7061	Draft	16	NZ_ABRX
0	Bacillus pumilus SAFR 032	Complete	1	NC_009848
0	Bacillus selenitireducens MLS10	Complete	1	NC_014219
0	Bacillus SG 1	Draft	186	NZ_ABCF
0	Bacillus subtilis 168	Complete	1	NC_000964
0	Bacillus subtilis 168	Draft	5	NZ_ABQK
0	Bacillus subtilis BSn5	Complete	1	NC_014976
0	Bacillus subtilis gtP20b	Draft	88	AEHM
0	Bacillus subtilis JH642	Draft	9	NZ_ABQM
0	Bacillus subtilis NCIB 3610	Draft	84	NZ_ABQL
0	Bacillus subtilis SMY	Draft	9	NZ_ABQN
0	Bacillus subtilis spizizenii ATCC 6633	Draft	37	NZ_ADGS
0	Bacillus subtilis spizizenii TU B 10	Complete	1	NC_016047
0	Bacillus subtilis spizizenii W23	Complete	1	NC_014479
0	Bacillus tusciae DSM 2912	Complete	1	NC_014098
0	Bacillus cereus R309803	Draft	227	NZ_ACLY
0	Bacillus mycoides Rock1 4	Draft	611	NZ_ACMV
0	Bacillus mycoides Rock3 17	Draft	311	NZ_ACMW
0	Bacillus pseudofirmus OF4	Complete	3	NC_013792, NC_013793, NC_013791
0	Bacillus pseudomycoides DSM 12442	Draft	305	NZ_ACMX
0	Bacillus cereus AH1271	Draft	367	NZ_ACMR
0	Bacillus cereus Rock1 3	Draft	171	NZ_ACMG
0	Bacillus cereus Rock3 28	Draft	1149	NZ_ACMI
0	Bacillus cereus Rock3 29	Draft	187	NZ_ACMJ
0	Bacillus cereus Rock3 44	Draft	1055	NZ_ACML
0	Bacillus cereus Rock4 18	Draft	176	NZ_ACMN
