# Supplementary material for: Gegenees: Fragmented Alignment of Multiple Genomes for Determining Phylogenomic Distances and Genetic Signatures Unique for Specified Target Groups
Source: PLoS One. 2012 Jun 18;7(6):e39107. doi: 10.1371/journal.pone.0039107 (PMC3377601; doi:10.1371/journal.pone.0039107)
Supplement: Table S4 — Templates and PCR results from the Bacillus spp. target groups T1–T5. The target groups are listed in Table S3. T1, T2, T3, T4 and T5 represent the target groups used to produce signatures and PCR-assays from. S1, S2 and S3 represent the three PCR-primer-pairs chosen from each of the five signatures. Field highlighted in green represents PCR-positive reaction, as expected from PCR-design. Field highlighted in orange represents PCR-negative reaction, as expected from PCR design. Field highlighted in red represents PCR-negative reaction, in contradiction to the positive results expected from PCR-design. (PDF) [file pone.0039107.s013.pdf]

Supplemental Table S4

Templates and PCR results from the *Bacillus* Spp. target groups T1-T5. The target groups are listed in Supplemental Table S3.

		T1			T2			T3			T4			T5		
**WGS**	**Strain name (culture collection ID of acquired strain)**	**S1**	**S2**	**S3**	**S1**	**S2**	**S3**	**S1**	**S2**	**S3**	**S1**	**S2**	**S3**	**S1**	**S2**	**S3**
wgs	Bacillus anthracis Vollum (NCTC 10340)	+	+	+	+	+	+	-	-	-	-	-	-	-	-	-
wgs	Bacillus cereus (BGSC 6E1)	-	-	-	+	+	+	-	-	-	-	-	-	-	-	-
wgs	Bacillus thuringiensis serovar andalousiensis (BGSC 4AW1)	-	-	-	+	+	+	-	-	-	-	-	-	-	-	-
wgs	Bacillus thuringiensis serovar monterrey (BGSC 4AJ1)	-	-	-	+	+	+	-	-	-	-	-	-	-	-	-
wgs	Bacillus thuringiensis serovar pondicheriensis (BGSC 4BA1)	-	-	-	+	+	+	-	-	-	-	-	-	-	-	-
wgs	Bacillus thuringiensis serovar pulsiensis (BGSC 4CC1)	-	-	-	+	+	+	-	-	-	-	-	-	-	-	-
-	Bacillus thuringiensis str. 97-27 (CIP 105674)	-	-	-	+	+	+	-	-	-	-	-	-	-	-	-
wgs	Bacillus cereus SJ1 (NRRL B-59452)	-	-	-	+	+	+	-	-	-	-	-	-	-	-	-
wgs	Bacillus cereus NVH0597-99	-	-	-	+	+	+	-	-	-	-	-	-	-	-	-
wgs	Bacillus cereus (ATCC 4342)	-	-	-	-	-	-	+	+	+	-	-	-	-	-	-
wgs	Bacillus thuringiensis serovar tochigiensis (BGSC 4Y1)	-	-	-	-	-	-	+	+	+	-	-	-	-	-	-
wgs	Bacillus thuringiensis serovar huazhongensis (BGSC 4BD1)	-	-	-	-	-	-	-	-	-	+	-	+	-	-	-
wgs	Bacillus thuringiensis serovar israelensis (ATCC 35646)	-	-	-	-	-	-	-	-	-	+	+	+	-	-	-
wgs	Bacillus cereus, ATCC 10876 (NCTC 7464)	-	-	-	-	-	-	-	-	-	+	-	+	-	-	-
wgs	Bacillus thuringiensis ATCC 10792 (DSM 2046)	-	-	-	-	-	-	-	-	-	+	+	+	-	-	-
wgs	Bacillus mycoides DSM 2048 (NCTC 12974)	-	-	-	-	-	-	-	-	-	-	-	-	+	-	+
-	Bacillus weihenstephanensis, KBAB4 (DSM 11821)	-	-	-	-	-	-	-	-	-	-	-	-	+	+	+
	Negative control	-	-	-	-	-	-	-	-	-	-	-	-	-	-	-

T1, T2, T3, T4 and T5 are the target groups used to produce signatures and PCR-assays from.

S1, S2 and S3 represent the three PCR-primer-pairs chosen from the five signatures.

Field highlighted in green represents PCR-positive reaction, as expected from PCR-design.

Field highlighted in orange represents PCR-negative reaction, as expected from PCR design.

Field highlighted in red represents PCR-negative reaction, in contradiction to the positive results expected from PCR-design
