# Supplementary material for: Gegenees: Fragmented Alignment of Multiple Genomes for Determining Phylogenomic Distances and Genetic Signatures Unique for Specified Target Groups
Source: PLoS One. 2012 Jun 18;7(6):e39107. doi: 10.1371/journal.pone.0039107 (PMC3377601; doi:10.1371/journal.pone.0039107)
Supplement: Table S5 — A list of Foot and Mouth disease Virus (FMDV) genomes used in the serotype comparisons. (PDF) [file pone.0039107.s014.pdf]

Supplemental Table S5

A list of Foot and Mouth disease Virus (FMDV) genomes used in the serotype comparisons.

Genome	State	No. of. subsequences/contigs	NCBI accession number
Footandmouth disease virus type Asia 1 Str vaccine IND 63 72	Complete	2	AY304994, NC_004915
Footandmouth disease virus O UKG 9443 2001 Str UKG 9443 2001	Complete	1	EF552695
Footandmouth disease virus type O Iso o1argentina iso5	Complete	1	AY593814
Footandmouth disease virus type A Iso IND 447 2005	Complete	1	HQ832583
Footandmouth disease virus type A Iso a16belem iso80	Complete	1	AY593756
Footandmouth disease virus type A Iso a10holland iso82	Complete	2	NC_011450, AY593751
Footandmouth disease virus type A Iso a22turkey iso66	Complete	1	AY593765
Footandmouth disease virus type O Iso WFL	Complete	1	EF175732
Footandmouth disease virus type O Iso MAY 8 2005	Complete	1	HQ632771
Footandmouth disease virus type O Iso UKG 417 2001	Complete	1	FJ542365
Footandmouth disease virus type Asia 1 Iso IND 116 90	Complete	1	DQ989305
Footandmouth disease virus type O Iso o11indonesia iso52	Complete	1	AY593813
Footandmouth disease virus type C Iso C S8p260p3d	Complete	1	DQ409185
Footandmouth disease virus type C Iso C S8p260d417	Complete	1	DQ409183
Footandmouth disease virus type O Str UKG 15101 2001	Complete	1	DQ404158
Footandmouth disease virus type A Iso a3mecklenburg iso81	Complete	1	AY593776
Footandmouth disease virus type O Iso UKG 2000 2001	Complete	1	FJ542369
Footandmouth disease virus type O Iso Israel 07 6389	Complete	1	FJ175664
Footandmouth disease virus type O Iso o10phil76 iso76	Complete	1	AY593812
Footandmouth disease virus type SAT 1 Iso sat1 20 iso11	Complete	1	AY593839
Footandmouth disease virus type O Str China 1 99Tibet	Complete	1	AF506822
Footandmouth disease virus type Asia 1 Iso MAY 9 99	Complete	1	HQ632774
Footandmouth disease virus type A Iso IND 437 2008	Complete	1	HQ832591
Footandmouth disease virus type O Str UKG 438 2001	Complete	1	DQ404174
Footandmouth disease virus O ES 2001 Iso O ES 2001	Complete	1	AY686687
Footandmouth disease virus type A Iso a22iraq 95 iso95	Complete	1	AY593762
Footandmouth disease virus type C Iso H595	Complete	1	AM409325
Footandmouth disease virus type O Str UKG 7675 2001	Complete	1	DQ404170
Footandmouth disease virus type A Iso a14 spain iso39	Complete	1	AY593754
Footandmouth disease virus type C Iso C S8p460d951	Complete	1	DQ409190
Footandmouth disease virus type Asia 1 Str ZB CHA 58att	Complete	1	DQ533483
Footandmouth disease virus type A Iso IND 88 2006	Complete	1	HQ832588
Footandmouth disease virus type O Str UKG 11 2001	Complete	1	DQ404180
Footandmouth disease virus type A Iso a13brazil iso75	Complete	1	AY593753
Footandmouth disease virus type Asia 1 Iso IND 423 01	Complete	1	DQ989319
Footandmouth disease virus type SAT 1 Iso sat1 1bech iso30	Complete	2	AY593838, NC_011451
Footandmouth disease virus type A Str IND17 82	Complete	1	HM854024
Footandmouth disease virus type SAT 1 Iso sat1 4srhod iso24	Complete	1	AY593841
Footandmouth disease virus type O Iso MAY 7 2001	Complete	1	HQ632769
Footandmouth disease virus type O Iso UKG 1558 2001	Complete	1	FJ542367
Footandmouth disease virus type O Iso PAK 44 2008	Complete	1	GU384682
Footandmouth disease virus type SAT 1 Iso sat1rhod iso33	Complete	1	AY593846
Footandmouth disease virus type O Iso otaiwan97 iso106 112	Complete	1	AY593835
Footandmouth disease virus type Asia 1 Iso asia1 1pak iso3	Complete	1	AY593795

Footandmouth disease virus type SAT 3 Iso sat3 3bech iso29	Complete	1	AY593851
Footandmouth disease virus type A Iso IND 249 2004	Complete	1	HQ832582
Footandmouth disease virus type SAT 2 Str unknown	Complete	1	AF540910 AF136607 AF283442 AF283459
Footandmouth disease virus type A Iso IND 64 2004	Complete	1	HQ832581
Footandmouth disease virus type O Iso Tibet CHA 99	Complete	1	AJ539138
Footandmouth disease virus type Asia 1 Str Asia1 WHN CHA 06	Complete	1	FJ906802
Footandmouth disease virus type O Iso Israel 07 6378	Complete	1	FJ175661
Footandmouth disease virus C3 Iso c3resende iso1	Complete	1	AY593807
Footandmouth disease virus type O Iso o2brescia iso17	Complete	1	AY593826
Footandmouth disease virus HKN 2002 Str HKN 2002	Complete	1	AY317098 AF525458 AY152808
Footandmouth disease virus type O Iso o1canefa iso59	Complete	1	AY593820
Footandmouth disease virus O UKG 4141 2001 Str UKG 4141 2001	Complete	1	EF552689
Footandmouth disease virus type Asia 1 Iso IND 182 02	Complete	1	DQ989320
Footandmouth disease virus type O Iso o1skr iso85	Complete	1	AY593824
Footandmouth disease virus type O Iso orey iran iso53	Complete	1	AY593834
Footandmouth disease virus type Asia 1 Iso IND 81 86	Complete	1	DQ989306
Footandmouth disease virus type Asia 1 Iso IND 61 02	Complete	1	DQ989318
Footandmouth disease virus type Asia 1 Iso IND 52 87	Complete	1	DQ989313
Footandmouth disease virus type O Iso o6pirbright iso58	Complete	1	AY593829
Footandmouth disease virus type C Iso C S8p360d951	Complete	1	DQ409187
Footandmouth disease virus type O Str UKG 9788 2001	Complete	1	DQ404166
Footandmouth disease virus type A Iso a5westerwald iso73	Complete	1	AY593781
Footandmouth disease virus type O Iso UKG 2640 2001	Complete	1	FJ542372
Footandmouth disease virus type O Str UKG 173 2001	Complete	1	DQ404175
Footandmouth disease virus type O Iso UKG 1734 2001	Complete	1	FJ542368
Footandmouth disease virus type O Str UKG 9327 2001	Complete	1	DQ404167
Footandmouth disease virus type A Str IND40 00	Complete	1	HM854025
Footandmouth disease virus O UKG 7039 2001 Str UKG 7039 2001	Complete	1	EF552690
Footandmouth disease virus type O Iso o10phil54 iso54	Complete	1	AY593811
Footandmouth disease virus type O Iso o3venezuela iso15	Complete	1	AY593827
Footandmouth disease virus type C Str C S8	Complete	2	NC_002554, AF274010
Footandmouth disease virus type C Iso C S8p360d417	Complete	1	DQ409186
Footandmouth disease virus type O Str UKG 14339 2001	Complete	1	DQ404163
Footandmouth disease virus type C Iso C S8p360p5d	Complete	1	DQ409188
Footandmouth disease virus type A Iso a27columbia iso78	Complete	1	AY593771
Footandmouth disease virus type C Iso C S8p260d999	Complete	1	DQ409184
Footandmouth disease virus type O Iso o7poland iso49	Complete	1	AY593830
Footandmouth disease virus type O Str UKG 220 2001	Complete	1	DQ404173
Footandmouth disease virus type C Str C S8p200	Complete	1	FJ824812
Footandmouth disease virus type A Iso a24 argentina iso9	Complete	1	AY593767
Footandmouth disease virus type Asia 1 Iso IND 82 96	Complete	1	DQ989309
Footandmouth disease virus type Asia 1 Iso IND 47 93	Complete	1	DQ989315
Footandmouth disease virus type A Iso IND 22 2006	Complete	1	HQ832584
Footandmouth disease virus type Asia 1 Iso IND 321 01	Complete	1	AY687333
Footandmouth disease virus type Asia 1 Iso asia1leb83 iso28	Complete	1	AY593800
Footandmouth disease virus type Asia 1 Iso IND 21 89	Complete	1	DQ989316
Footandmouth disease virus type O Iso UKG 35 2001	Complete	1	AJ539141
Footandmouth disease virus type Asia 1 Iso Asia1 YS CHA 05	Complete	1	GU931682

Footandmouth disease virus type SAT 1 Iso sat1 3swa iso14	Complete	1	AY593840
Footandmouth disease virus type O Iso o1campos iso96	Complete	1	AY593818
Footandmouth disease virus type Asia 1 Iso asia1 2isrl3 63 iso6	Complete	1	AY593796
Footandmouth disease virus type O Str UKG 14603 2001	Complete	1	DQ404159
Footandmouth disease virus type O Str UKG 14524 2001	Complete	1	DQ404160
Footandmouth disease virus type A Iso a2spain iso7	Complete	1	AY593774
Footandmouth disease virus type Asia 1 Iso IND 13 91	Complete	1	DQ989312
Footandmouth disease virus O UKG 9161 2001 Str UKG 9161 2001	Complete	1	EF552691
Footandmouth disease virus O UKG 4998 2001 Str UKG 4998 2001	Complete	1	EF552694
Footandmouth disease virus type SAT 1 Iso sat1 6swa iso16	Complete	1	AY593843
Footandmouth disease virus type Asia 1 Iso IND 354 01	Complete	1	DQ989314
Footandmouth disease virus type O Iso UKG 2526 2001	Complete	1	FJ542371
Footandmouth disease virus type A Iso IND 161 2003	Complete	1	HQ832578
Footandmouth disease virus type A Iso a18zulia iso40	Complete	1	AY593758
Footandmouth disease virus type A Iso a25 argentina iso38	Complete	1	AY593769
Footandmouth disease virus C3 Iso c3ind iso19	Complete	1	AY593806
Footandmouth disease virus type Asia 1 Iso asia1leb4 iso4	Complete	1	AY593799
Footandmouth disease virus type O Str UKG 14391 2001	Complete	1	DQ404161
Footandmouth disease virus type O Str UKG 4569 2001	Complete	1	DQ404171
Footandmouth disease virus type O Str UKG 8098 2001	Complete	1	EU214601
Footandmouth disease virus type A Iso IND 21 1990	Complete	1	HQ832576
Footandmouth disease virus type O Iso openghu iso108	Complete	1	AY593833
Footandmouth disease virus type O Str UKG 621 2001	Complete	1	DQ404172
Footandmouth disease virus type A Iso aparma iso55	Complete	1	AY593792
Footandmouth disease virus type Asia 1 Str Asia1 MOG 05	Complete	1	EF614458
Footandmouth disease virus C1 Iso c1noville iso56	Complete	1	AY593804
Footandmouth disease virus type A Iso a1bayern iso41	Complete	1	AY593759
Footandmouth disease virus type O Iso O NY00	Complete	1	AY333431
Footandmouth disease virus type O Iso o1brugge iso79	Complete	1	AY593817
Footandmouth disease virus type O Str Akesu 58	Complete	1	AF511039
Footandmouth disease virus type A Iso a4 W Germany iso42	Complete	1	AY593777
Footandmouth disease virus type O Str UKG 11676 2001	Complete	1	DQ404164
Footandmouth disease virus type A Iso a20ussr iso10	Complete	1	AY593760
Footandmouth disease virus type A Iso abrazil iso67	Complete	1	AY593788
Footandmouth disease virus O UKG 5470 2001 Str UKG 5470 2001	Complete	1	EF552696
Footandmouth disease virus type SAT 2 Iso sat2 2 iso25	Complete	1	AY593848
Footandmouth disease virus type SAT 1 Iso sat1bot iso47	Complete	1	AY593845
Footandmouth disease virus type O Iso MAY 3 2000	Complete	1	HQ632768
Footandmouth disease virus type A Iso acanefa iso48	Complete	1	AY593789
Footandmouth disease virus type O Str OGBF15	Complete	1	DQ478936
Footandmouth disease virus type A Iso a28 Turkey iso44	Complete	1	AY593772
Footandmouth disease virus type A Iso aargp64 iso100	Complete	1	AY593785
Footandmouth disease virus type O Str UKG 126 2001	Complete	1	DQ404179
Footandmouth disease virus type A Iso a argentina 2000 iso104	Complete	1	AY593782
Footandmouth disease virus type O Iso o1bfs46 iso46	Complete	1	AY593816
Footandmouth disease virus type C Iso C S8p460d417	Complete	1	DQ409189
Footandmouth disease virus type Asia 1 Iso IND 101 99	Complete	1	DQ989310
Footandmouth disease virus type A Iso a4spain iso62	Complete	1	AY593778

Footandmouth disease virus O SKR 2000 Str unknown	Complete	1	AF377945
Footandmouth disease virus type Asia 1 Iso IND 334 00	Complete	1	DQ989304
Footandmouth disease virus type A Iso A uruguay 2001 iso98	Complete	1	AY593802
Footandmouth disease virus type A Iso IND 245 2007	Complete	1	HQ832590
Footandmouth disease virus type O Iso PAK 45 2008	Complete	1	GU384683
Footandmouth disease virus type O Iso SKR 2000	Complete	1	AJ539139
Footandmouth disease virus type Asia 1 Str YNBS 58	Complete	1	AY390432
Footandmouth disease virus type O Iso ouk2001x iso84	Complete	1	AY593836
Footandmouth disease virus type O Iso MAY 1 2004	Complete	1	HQ632770
Footandmouth disease virus type O Str UKG 14476 2001	Complete	1	DQ404162
Footandmouth disease virus type SAT 2 Iso sat2 3kenya 21	Complete	2	NC_003992, AY593849
Footandmouth disease virus type A Iso a12valle 119 iso20	Complete	1	AY593752
Footandmouth disease virus type Asia 1 Iso IND 139 02	Complete	1	DQ989322
Footandmouth disease virus type O Str OGBF15 derivative	Complete	1	DQ478937
Footandmouth disease virus type A Iso a22iraq64 iso86	Complete	1	AY593763
Footandmouth disease virus type Asia 1 Iso IND 247 92	Complete	1	DQ989307
Footandmouth disease virus type O Iso UKG 2085 2001	Complete	1	FJ542370
Footandmouth disease virus type A Iso a4wg iso72	Complete	1	AY593779
Footandmouth disease virus type A Iso a32ven iso36	Complete	1	AY593775
Footandmouth disease virus O UKG 5681 2001 Str UKG 5681 2001	Complete	1	EF552697
Footandmouth disease virus O UKG 3952 2001 Str UKG 3952 2001	Complete	1	EF552688
Footandmouth disease virus type A Iso a general lopez iso102	Complete	1	AY593790
Footandmouth disease virus type O Str UKG 150 2001	Complete	1	DQ404176
Footandmouth disease virus type O Iso TAW 2 99 BOV	Complete	1	AJ539137
Footandmouth disease virus type A Iso avenceslau iso70	Complete	1	AY593803
Footandmouth disease virus type O Str China 5 99Fujian	Complete	1	HQ009509
Footandmouth disease virus type A Iso a17 Aguarulbos iso83	Complete	1	AY593757
Footandmouth disease virus type A Iso airan iso105	Complete	1	AY593791
Footandmouth disease virus type A Iso IND 110 1999	Complete	1	HQ832577
Footandmouth disease virus type O Iso MAY 7 2007	Complete	1	HQ632772
Footandmouth disease virus type Asia 1 Iso asia1 3kimron iso61	Complete	1	AY593797
Footandmouth disease virus C1 Iso c1ober iso88	Complete	1	AY593805
Footandmouth disease virus type O Iso Israel 07 6391	Complete	1	FJ175665
Footandmouth disease virus type A Str IND17 77	Complete	1	HM854022
Footandmouth disease virus type A Iso a29peru iso37	Complete	1	AY593773
Footandmouth disease virus type A Iso MAY 3 2007	Complete	1	HQ632773
Footandmouth disease virus type O Iso ouruguay 51 iso51	Complete	1	AY593837
Footandmouth disease virus type A Iso IND 281 2003	Complete	1	HQ832579
Footandmouth disease virus type A Iso IND 818 2003	Complete	1	HQ832580
Footandmouth disease virus type SAT 1 Iso sat1 5sa iso13	Complete	1	AY593842
Footandmouth disease virus type O Str O YM YN 2000	Complete	1	HQ412603
Footandmouth disease virus type O Str unknown	Complete	2	AF308157, NC_004004
Footandmouth disease virus C4 Iso C4 Tierra del Fuego iso2	Complete	1	AY593808
Footandmouth disease virus type A Iso IND 26 2006	Complete	1	HQ832585
Footandmouth disease virus type Asia 1 Iso IND 151 94	Complete	1	DQ989303
Footandmouth disease virus type O Iso o1manisa iso87	Complete	1	AY593823
Footandmouth disease virus type O Iso o1bfs iso18	Complete	1	AY593815
Footandmouth disease virus type O Str UKG 7038 2001	Complete	1	DQ404169

Footandmouth disease virus type A Iso aphilippines iso50	Complete	1	AY593793
Footandmouth disease virus type O Iso O SKR 2000	Complete	2	AY312587, AY312586
Footandmouth disease virus type Asia 1 Iso IND 438 01	Complete	1	DQ989321
Footandmouth disease virus type O Str Chu Pei	Complete	1	AF026168
Footandmouth disease virus type A Iso IND 109 2006	Complete	1	HQ832589
Footandmouth disease virus type A Iso asabana iso68	Complete	1	AY593794
Footandmouth disease virus type O Str O YS CHA 05	Complete	1	HM008917
Footandmouth disease virus type Asia 1 Iso asia1leb 89 iso89	Complete	1	AY593798
Footandmouth disease virus type O Iso o1campos94 iso94	Complete	1	AY593819
Footandmouth disease virus type A Iso aargp55 iso99	Complete	1	AY593784
Footandmouth disease virus type SAT 3 Iso sat3 3kenya iso22	Complete	1	AY593852
Footandmouth disease virus type A Iso Lindholm 13PAK3 2006	Complete	1	EF117837
Footandmouth disease virus type O Iso o1valle iso64	Complete	1	AY593825
Footandmouth disease virus type O Str UKG 9964 2001	Complete	1	DQ404165
Footandmouth disease virus type A Iso abage iso63	Complete	1	AY593787
Footandmouth disease virus type A Iso IND 43 2006	Complete	1	HQ832586
Footandmouth disease virus type O Iso O SKR 2002	Complete	2	AY312589, AY312588
Footandmouth disease virus type A Iso a24cruzeiro iso71	Complete	1	AY593768
Footandmouth disease virus type O Iso SAR 19 2000	Complete	1	AJ539140
Footandmouth disease virus type A Iso a21kenya iso77	Complete	1	AY593761
Footandmouth disease virus type SAT 1 Iso sat1 7isrl iso12	Complete	1	AY593844
Footandmouth disease virus type O Iso Israel 07 6382	Complete	1	FJ175663
Footandmouth disease virus type O Iso TAW 2 99 TC	Complete	1	AJ539136
Footandmouth disease virus type O Iso O UK2001 ED	Complete	1	AY593831
Footandmouth disease virus type Asia 1 Iso IND 37 02	Complete	1	DQ989311
Footandmouth disease virus type O Str UKG 128 2001	Complete	1	DQ404177
Footandmouth disease virus type C Iso C S8p460p5d	Complete	1	DQ409191
Footandmouth disease virus type O Iso O UK2001 FB	Complete	1	AY593832
Footandmouth disease virus type SAT 2 Iso sat2 1rhod iso26	Complete	1	AY593847
Footandmouth disease virus type O Iso HKN 20 2010	Complete	1	HM229661
Footandmouth disease virus type O Iso o1m11 iso57	Complete	1	AY593822
Footandmouth disease virus type O Iso lz	Complete	1	DQ248888
Footandmouth disease virus type A Iso IND 17 2009	Complete	1	HQ832592
Footandmouth disease virus type A Iso IND 50 2006	Complete	1	HQ832587
Footandmouth disease virus type O Iso o1caseros iso35	Complete	1	AY593821
Footandmouth disease virus type Asia 1 Iso IND 397 97	Complete	1	DQ989308
Footandmouth disease virus type A Iso a15thailand iso43	Complete	1	AY593755
Footandmouth disease virus type A Iso aarg Trenquelauquen iso103	Complete	1	AY593786
Footandmouth disease virus O UKG 4014 2001 Str UKG 4014 2001	Complete	1	EF552693
Footandmouth disease virus type A Iso a26arg iso74	Complete	1	AY593770
Footandmouth disease virus type Asia 1 Str Asia 1 Jiangsu China 2005	Complete	1	EF149009
Footandmouth disease virus type O Iso UKG 1450 2001	Complete	1	FJ542366
Footandmouth disease virus type O Str O SKR 14 02	Complete	1	EF614457
Footandmouth disease virus type O Iso Israel 07 6380	Complete	1	FJ175662
Footandmouth disease virus type O Iso O1Campos	Complete	1	AJ320488
Footandmouth disease virus type O Str UKG 127 2001	Complete	1	DQ404178
Footandmouth disease virus type C Iso cwald iso32	Complete	1	AY593810
Footandmouth disease virus type Asia 1 Str IND 491 97; WBN 117 85	Complete	1	AY687334

Footandmouth disease virus type Asia 1 Iso IND 148 01	Complete	1	DQ989317
Footandmouth disease virus type A Str IND258 99	Complete	1	HM854023
Footandmouth disease virus O UKG 7299 2001 Str UKG 7299 2001	Complete	1	EF552692
Footandmouth disease virus type A Iso A30 Uruguay 68 iso90	Complete	1	AY593801
Footandmouth disease virus type A Iso a5allier iso45	Complete	1	AY593780
Footandmouth disease virus type A Iso aarg2001 iso93	Complete	1	AY593783
Footandmouth disease virus type A Str IND81 00	Complete	1	HM854021
Footandmouth disease virus type O Str UKG 9011 2001	Complete	1	DQ404168
Footandmouth disease virus type O Str OMIII	Complete	1	AY359854
Footandmouth disease virus C5 Iso c5arg iso60	Complete	1	AY593809
Footandmouth disease virus type O Iso o5india iso34	Complete	1	AY593828
Footandmouth disease virus type SAT 3 Iso sat3 4bech iso23	Complete	1	AY593853
Footandmouth disease virus type Asia 1 Str Asia 1 HNK CHA 05	Complete	1	EF149010
Footandmouth disease virus type A Iso a22iraq70 iso92	Complete	1	AY593764
Footandmouth disease virus type O Iso Israel 07 6387	Complete	1	FJ175666
Footandmouth disease virus type SAT 3 Iso sat3 2sa iso27	Complete	2	AY593850, NC_011452
Footandmouth disease virus type A Iso a23kenya iso8	Complete	1	AY593766
Footandmouth disease virus type Asia 1 Iso IND 97 03	Complete	1	DQ989323
