# Supplementary material for: Gegenees: Fragmented Alignment of Multiple Genomes for Determining Phylogenomic Distances and Genetic Signatures Unique for Specified Target Groups
Source: PLoS One. 2012 Jun 18;7(6):e39107. doi: 10.1371/journal.pone.0039107 (PMC3377601; doi:10.1371/journal.pone.0039107)
Supplement: Table S6 — A list of Escherichia spp. genomes used in the Escherichia comparison. (PDF) [file pone.0039107.s015.pdf]

Supplemental Table S6

A list of *Escherichia* Spp. genomes used in the *Escherichia* comparison.

Genome	State	No. of. subsequences/contigs	NCBI accession number
Escherichia coli K 12 substr MG1655	Complete	1	NC_000913
Escherichia coli MS 187 1	Draft	310	NZ_ADTQ
Escherichia coli MS 116 1	Draft	484	NZ_ADTZ
Escherichia coli 2362 75	Draft	83	NZ_ADUL
Escherichia coli O157 H7 EC508	Draft	272	NZ_ABHW
Escherichia coli MS 124 1	Draft	176	NZ_ADWT
Escherichia coli O157 H7 EC4042	Draft	4	NZ_ABHM
Escherichia coli MS 78 1	Draft	410	NZ_ADTY
Escherichia coli MS 196 1	Draft	785	NZ_ADUD
Escherichia coli S88	Complete	2	NC_011742, NC_011747
Escherichia coli B354	Draft	69	ACXG
Escherichia coli O157 H7 Sakai	Complete	3	NC_002127, NC_002695, NC_002128
Escherichia coli O157 H7 EC4045	Draft	8	NZ_ABHL
Escherichia coli MS 69 1	Draft	425	NZ_ADTP
Escherichia coli ATCC 8739	Complete	1	NC_010468
Escherichia coli 1827 70	Draft	35	NZ_ADUK
Escherichia coli MS 115 1	Draft	423	NZ_ADTL
Escherichia coli O157 H7 TW14588	Draft	10	NZ_ABKY
Escherichia coli CFT073	Complete	1	NC_004431
Escherichia coli K 12 substr DH10B	Complete	1	NC_010473
Escherichia coli B REL606	Complete	1	NC_012967
Escherichia coli M718	Draft	188	ADAW
Escherichia coli MS 21 1	Draft	540	NZ_ADTR
Escherichia coli W	Draft	88	NZ_AEDF
Escherichia coli MS 145 7	Draft	143	NZ_ADWS
Escherichia coli TA206	Draft	187	ADAX
Escherichia coli OP50	Draft	2939	NZ_ADBT
Escherichia coli O157 H7 FRIK2000	Draft	247	NZ_ACXO
Escherichia 3 2 53FAA	Draft	173	ACAC
Escherichia coli O157 H7 EDL933	Complete	2	NC_007414, NC_002655
Escherichia coli MS 107 1	Draft	72	NZ_ADWV
Escherichia coli O26 H11 11368	Complete	5	NC_013361, NC_014543, NC_013363, NC_013369, NC_013362
Escherichia coli O157 H7 EC4486	Draft	165	NZ_ABHS
Escherichia coli TA271	Draft	191	ADAZ
Escherichia coli H299	Draft	229	ADBC
Escherichia coli MS 198 1	Draft	564	NZ_ADTJ
Escherichia coli IAI1	Complete	1	NC_011741
Escherichia fergusonii ATCC 35469	Complete	2	NC_011740, NC_011743
Escherichia coli O157 H7 EC4076	Draft	135	NZ_ABHQ
Escherichia coli O157 H7 EC4501	Draft	250	NZ_ABHT
Escherichia coli O157 H7 EC4401	Draft	186	NZ_ABHR
Escherichia coli 101 1	Draft	91	NZ_AAMK
Escherichia coli O127 H6 E2348 69	Complete	3	NC_011603, NC_011601, NC_011602
Escherichia coli O55 H7 CB9615	Complete	2	NC_013941, NC_013942

Escherichia coli MS 182 1	Draft	456	NZ_ADTM
Escherichia coli MS 146 1	Draft	355	NZ_ADTN
Escherichia coli MS 119 7	Draft	130	NZ_ADWU
Escherichia coli E24377A	Complete	7	NC_009789, NC_009786, NC_009801, NC_009788, NC_009787, NC_009790, NC_009791
Escherichia coli O157 H7 EC869	Draft	147	NZ_ABHU
Escherichia coli MS 185 1	Draft	378	NZ_ADUE
Escherichia coli O157 H7 EC4024	Draft	364	NZ_ABJT
Escherichia coli MS 84 1	Draft	432	NZ_ADTK
Escherichia albertii TW07627	Draft	64	NZ_ABKX
Escherichia coli O157 H7 EC4206	Draft	7	NZ_ABHK
Escherichia coli FVEC1302	Draft	149	ACXH
Escherichia coli B088	Draft	148	ACXE
Escherichia coli NC101	Draft	27	NZ_AEFA
Escherichia coli APEC O1	Complete	3	NC_008563, NC_009837, NC_009838
Escherichia coli H591	Draft	190	ADBB
Escherichia coli TA280	Draft	168	ADBA
Escherichia coli B185	Draft	115	ACXF
Escherichia coli UMN026	Complete	3	NC_011739, NC_011749, NC_011751
Escherichia coli H736	Draft	135	ADAU
Escherichia coli BW2952	Complete	1	NC_012759
Escherichia coli SMS 3 5	Complete	5	NC_010488, NC_010487, NC_010485, NC_010486, NC_010498
Escherichia coli O103 H2 12009	Complete	2	NC_013353, NC_013354
Escherichia coli 53638	Draft	2	NZ_AAKB
Escherichia coli UTI89	Complete	2	NC_007941, NC_007946
Escherichia coli MS 45 1	Draft	396	NZ_ADTO
Escherichia coli 536	Complete	1	NC_008253
Escherichia coli B7A	Draft	289	NZ_AAJT
Escherichia coli 83972	Draft	159	NZ_ACGN
Escherichia coli 55989	Complete	1	NC_011748
Escherichia coli O157 H7 TW14359	Complete	2	NC_013010, NC_013008
Escherichia coli O111 H 11128	Complete	6	NC_013364, NC_013365, NC_013368, NC_013367, NC_013366, NC_013370
Escherichia coli O157 H7 FRIK966	Draft	316	NZ_ACXN
Escherichia coli BL21 Gold DE3 pLysS AG	Complete	1	NC_012947
Escherichia coli FVEC1412	Draft	137	ACXI
Escherichia coli E22	Draft	127	NZ_AAJV
Escherichia coli O157 H7 EC4113	Draft	231	NZ_ABHP
Escherichia coli ED1a	Complete	1	NC_011745
Escherichia coli O157 H7 EC4115	Complete	3	NC_011350, NC_011353, NC_011351
Escherichia coli O157 H7 EC4196	Draft	186	NZ_ABHO
Escherichia coli MS 175 1	Draft	413	NZ_ADUB
Escherichia coli IAI39	Complete	1	NC_011750
Escherichia 4 1 40B	Draft	126	NZ_ACDM
Escherichia coli TA143	Draft	168	ADAY
Escherichia coli SE11	Complete	7	NC_011416, NC_011415, NC_011419, NC_011408, NC_011411, NC_011413, NC_011407
Escherichia coli B171	Draft	262	NZ_AAJX
Escherichia coli MS 200 1	Draft	445	NZ_ADUC
Escherichia coli M605	Draft	162	ADAV
Escherichia coli HS	Complete	1	NC_009800

Escherichia 1 1 43	Draft	91	ACID
Escherichia coli F11	Draft	119	NZ_AAJU
Escherichia coli E110019	Draft	137	NZ_AAJW
Escherichia coli O104 H4 str 01 09591	Draft	287	AFPS
Escherichia coli O104 H4 str LB226692	Draft	356	AFOB
