# Supplementary material for: Gegenees: Fragmented Alignment of Multiple Genomes for Determining Phylogenomic Distances and Genetic Signatures Unique for Specified Target Groups
Source: PLoS One. 2012 Jun 18;7(6):e39107. doi: 10.1371/journal.pone.0039107 (PMC3377601; doi:10.1371/journal.pone.0039107)
Supplement: Table S7 — A list of Escherichia strains and plasmids used in the Escherichia plasmid comparison. (PDF) [file pone.0039107.s016.pdf]

**Supplemental Table S7**

**A list of *Escherichia* Spp. plasmids used in the *Escherichia* comparison.**

| Genome | State | No. of. subsequences/contigs | NCBI accession number |
|---|---|---|---|
| Escherichia coli O104 H4 str 01 09591 | Draft | 287 | AFPS |
| Escherichia coli O104 H4 str LB226692 | Draft | 356 | AFOB |
| pAPEC-O1-R | Complete | 1 | NC_009838 |
| pR751 | Complete | 1 | NC_001735 |
| pEK204 | Complete | 1 | EU935740 |
| pMT-1 | Complete | 1 | AF074611 |
| pK29 | Complete | 1 | EF382672 |
| pOLA52 | Complete | 1 | EU370913 AY241669 AY835616 |
| pCTXM360 | Complete | 1 | NC_011641 |
| pC15-1a | Complete | 1 | AY458016 |
| pEK516 | Complete | 1 | EU935738 |
| pSERB1 | Complete | 1 | AY686591 |
| pR46 | Complete | 1 | AY046276 AF117344 |
| pHCM1 | Complete | 1 | NC_003384 |
| pEC L8 | Complete | 1 | GU371928 |
| pR621a | Complete | 1 | AP011954 |
| pMAR7 | Complete | 1 | DQ388534 |
| pAA O42 | Complete | 1 | FN554767 |
| pO103 | Complete | 1 | NC_013354 |
| pCTX M3 | Complete | 1 | AF550415 |
| pEC B24 | Complete | 1 | GU371926 |
| pETEC 6 | Complete | 1 | NC_009789 |
| pR27 | Complete | 1 | AF250878 |
| pR7K | Complete | 1 | NC_010643 |
| pMUR050 | Complete | 1 | AY522431 |
| pCoo | Complete | 1 | NC_007635 |
| pKP96 | Complete | 1 | EU195449 |
| pO157 | Complete | 1 | AF074613 |
| pUTI89 | Complete | 1 | NC_007941 |
| p55989 | Complete | 1 | NC_011752 |
| pJP4 | Complete | 1 | NC_005912 |
| pEC Bactec | Complete | 1 | GU371927 |

| | | | |
|---|---|---|---|
| pEC4115 | Complete | 1 | NC_011351 |
| pEC L46 | Complete | 1 | GU371929 |
| pEK499 | Complete | 1 | EU935739 |
| pB171 | Complete | 1 | AB024946 |
| pWR501 | Complete | 1 | AF348706 |
| pR478 | Complete | 1 | BX664015 |
| pEL60 | Complete | 1 | NC_005246 |
| pEntH10407 | Complete | 1 | NC_013507 |
| p1081 | Complete | 1 | NC_014232 |
