# Supplementary material for: Gegenees: Fragmented Alignment of Multiple Genomes for Determining Phylogenomic Distances and Genetic Signatures Unique for Specified Target Groups
Source: PLoS One. 2012 Jun 18;7(6):e39107. doi: 10.1371/journal.pone.0039107 (PMC3377601; doi:10.1371/journal.pone.0039107)
Supplement: Text S1 — In-depth material and methods. Text describing the structure of files used/created by Gegenees, the workflow and settings of the BLAST runs and also the PCR setup. (PDF) [file pone.0039107.s017.pdf]

Supplemental Material and Methods

Gegenees Workspace Organization

Gegenees uses a 'workspace' that contains comparisons. Each comparison is represented by a directory with the prefix 'comparison_'. A default database is always present in the directory 'database'. Custom databases are represented by directories with the prefix 'database_'. The ftp settings and translation tables are stored in the 'ftp' directory. Each ftp site is stored as a '.site' file. Custom ftp sites can be set up by making new modified '.site' files. If needed, genomes are unzipped during the download process. Each genome, in a database or in a comparison, is represented by a directory in the form '<genome name>--<type>--' where type can be 'Draft' or 'Complete'. The genome directories can contain one or several GenBank formatted files, each containing one or several sequences. The GenBank files must end with '.gbk' or '.gbff'. A Comparison directory contains genome directories and alignment directories with the prefix 'alignment_'. The Comparison directory also contains a file 'comparison.geg' that stores information about the comparison.

The Alignment

When an alignment is created, the genomes are copied into the alignment directory. Each genome will end up in a file with the systematic naming GNr (i.e. G0, G1, G2...). The translation is stored in the 'alignment.geg' file. When an alignment is started, the 'GNr.gbk' file is converted to 'GNr.fasta' and the fragments are created in the 'GNr.frag'. Information about fragment coordinates is stored in 'GNr.fraginfo'. All BLAST commands for the analysis are generated during startup and stored in the 'blastcontrol.ss' file. To get a better estimation of the alignment time (and time left), the 'blastcontrol.ss' is shuffled and stored in 'blastcontrol.ss.runorder'. The commands in the '.runorder' file are executed sequentially yielding a 'GNr_GNr.result' file. When the BLAST job is complete, a 'GNr_GNr.result.done' file is created. If the program is paused, interrupted or crashes, the comparison can be continued since all comparisons that have generated a .done file are skipped during the resume process. If an expert user wants to divide the comparison among several computers, several copies of the alignment can be created and the '.runorder' file modified so that different parts are run on different machines. The '.result' files and the '.result.done' files can then be combined for the final step of the analysis. Finally, the similarity matrices (prefix 'Heat_') and the first level-biomarker tables (prefix 'Bmark_') are created. The '.done' files are removed and the result files are packed into result tables 'GNr.blastresults'. In the Alignment dir, a 'genome.info' file that contains information from the genomes in the alignment, is stored. Sort-orders for the heat-plot are stored in files with the prefix 'Sort_'. There is also a 'GNr.geg' file with information about the subsequences of each genome and a 'GNr.fragAnnot' file that contains annotations of the fragments. In the alignment directory there are also analysis directories that contain an 'analysis.geg' file. This file contains the grouping settings and the second level biomarker file that is

recalculated to fit the current target group settings. The default analysis is stored in the 'analysis' directory and others have the prefix 'analysis_'. When a alignment is updated, the packed result files are unpacked and the '.done' files are recreated. Then the 'blastcontrol.ss' file and the 'blastcontrol.ss.runorder' are updated with the missing commands after which the alignment can be resumed.

The fragmentation

Fragments are created by a sliding window approach. The first fragment is created from the first nucleotide and the window is moved with the step-size. When the last fragment that can be fitted into the sequence has been made, a short stretch of nucleotides may be left. Therefore a final fragment is created fitting the absolute end of the sequence. If a contig in a draft genome is shorter than the fragment size, a warning is issued and the contig is excluded. If a fragment contains only 'n', BLAST will issue an error and therefore a final 'a' is added to such fragments. Likewise, if in TBLASTX mode no full codon is present in an 'n' rich fragment, a final 'aaa' is added.

The BLAST control engine

During our calculation optimization, we came to the conclusion that BLAST comparisons are most efficient if they are run in parallel threads. The number of threads should be at least as many as the number of cores in the processor. Gegenees asks the system how many cores are available and then runs the BLAST commands in that many parallel threads. BLAST is run with the Dust and SEG filter turned off and with the tabular output option on. If a primer alignment is used, the word size is set to 7. When a BLAST job is completed the result-file is filtered and everything except the score of the best hit is discarded. If a fragment gets no hit, the score is set to 0. Since the filtering is done continuously during the analysis, huge disk space usage is avoided.

Similarity matrices and biomarker tables

Similarity matrices are done by normalizing the scores towards the maximal possible score. For a BLASTN comparison, this is defined as the overall maximum score in the self-comparison. When using TBLASTX, the score is dependent on the sequence composition. Therefore the maximum score in the self-comparison for that particular fragment is used. A set of predefined thresholds is used to create a number of average values. The biomarker tables contain the data on how every fragment scores with respect to the different genomes and these are used to calculate the biomarker scores displayed in the program.

Dendrogram production

The similarity matrices in the Gegenees heat-plots are converted into distance matrices and exported in the Nexus file format. Trees were created using SplitsTree4 [1] by the use of the neighbor joining method and

trees were drawn in the phylogram-form. In all figures shown in this article, the strain at the bottom of the tree was used as out-group.

Implementation

Gegenees was written in JAVA in eclipse using the Rich Client Platform (RCP) framework. The program is free for non-profit and academic usage. Questions regarding access to source code should be addressed to the corresponding author.

PCR reactions

Primers targeting the regions defined by Gegenees were designed using Primer3 software [2]. Real-time PCR was performed using a 7500 Fast Real-Time PCR System (Applied Biosystems, Life Technologies Corp., Carlsbad, CA, USA) with PerfeCTa SYBR Green SuperMix (Quanta Biosciences Inc., Gaithersburg, MD, USA) and ROX as a passive reference. The primers used were: T1S1f 5'-ATATGGCTGATGCGCCTAAA-3', T1S1r 5'-AATAATTGCACCGCCAAGAG-3', T1S2f 5'-TTATGCTTTGCGCCTACAGA-3', T1S2r 5'-CATCTCGCGTTTCTTGATGA-3', T1S3f 5'-TGGAGCCGCAATTGAATTAT-3', T1S3r 5'-TGCCGTTCTATTCCTTCCTG-3',
T2S1f 5'-CCATCTTAACTTCGGTCCACA-3', T2S1r 5'-GGTTGATTTTGGTTAGGGTGAA-3', T2S2f 5'-TTCCCACCCTTCTTTTAACAC-3', T2S2r 5'-CAGAGGTTCTTTCTAGCGTTTT-3', T2S3f 5'-CACGTTCTTTATCTCCAACCAA-3', T2S3r 5'-TCCGCATTACTTAGCCGTAAA-3',
T3S1f 5'-TTATCTATCCCGCCGTTGGT-3', T3S1r 5'-AAATAGCTTCGCCACTTCCT-3', T3S2f 5'-GCTGTATTGGCAGGACATCG-3', T3S2r 5'-AGCAGTGTAACTTTGTCTTTTCCTT-3', T3S3f 5'-AAAATGTGTGAAAGGACGAATG-3', T3S3r 5'-CCCCCTATACTTGTCGCCTA-3',
T4S1f 5'-AAGCGCTTCGCACATTATCT-3', T4S1r 5'-CTCCGCTATGGTAGGCTTTG-3', T4S2f 5'-AAACGAAGACAGGGGAAATG-3', T4S2r 5'-CTGCAAAAACATGGTCCTCA-3', T4S3f 5'-AAGTGTGCAGGAGTTAGAGTTGC-3', T4S3r 5'-GCTTCTTGCTCATTTACTTTTTCA-3',
T5S1f 5'-TTCCTTTTTGGAACCCATTG-3', T5S1r 5'-AGGTTTCACCCAATCTGCTG-3', T5S2f 5'-TACGATTGATGCCTCGTTGA-3', T5S2r 5'-GGGTATCCATCCCCACTGAT-3', T5S3f 5'-CGCCTTGATTTAGGGGTTTT-3', T5S3r 5'-CCCCCATACGTAAACATTCC-3', where the number after the 'T' represents target group number.

Templates were DNA extracted using the MasterPure™ Gram Positive DNA Purification Kit (Epicentre Biotechnologies, Madison, WI, USA) from strains listed in Supplemental Table S4. The following cycle parameters were used: 1 cycle of 95° for 5 min, 40 cycles of 95° for 15 s, 60° for 45 s followed by a melting point analysis. Analysis was carried out using the 7500 Software v2.0.4.

1. Huson DH, Bryant D (2006) Application of phylogenetic networks in evolutionary studies. Molecular biology and evolution 23: 254-267.

2. Rozen S, Skaletsky H (2000) Primer3 on the WWW for general users and for biologist programmers. Methods in molecular biology 132: 365-386.
