# Supplementary material for: Gegenees: Fragmented Alignment of Multiple Genomes for Determining Phylogenomic Distances and Genetic Signatures Unique for Specified Target Groups
Source: PLoS One. 2012 Jun 18;7(6):e39107. doi: 10.1371/journal.pone.0039107 (PMC3377601; doi:10.1371/journal.pone.0039107)
Supplement: Figure S2 — Average whole genome similarity of the Bacillus -genus. A heat-plot showing the similarity matrix when comparing 134 Bacillus spp. genomes with Gegenees. (PDF) [file pone.0039107.s003.pdf]

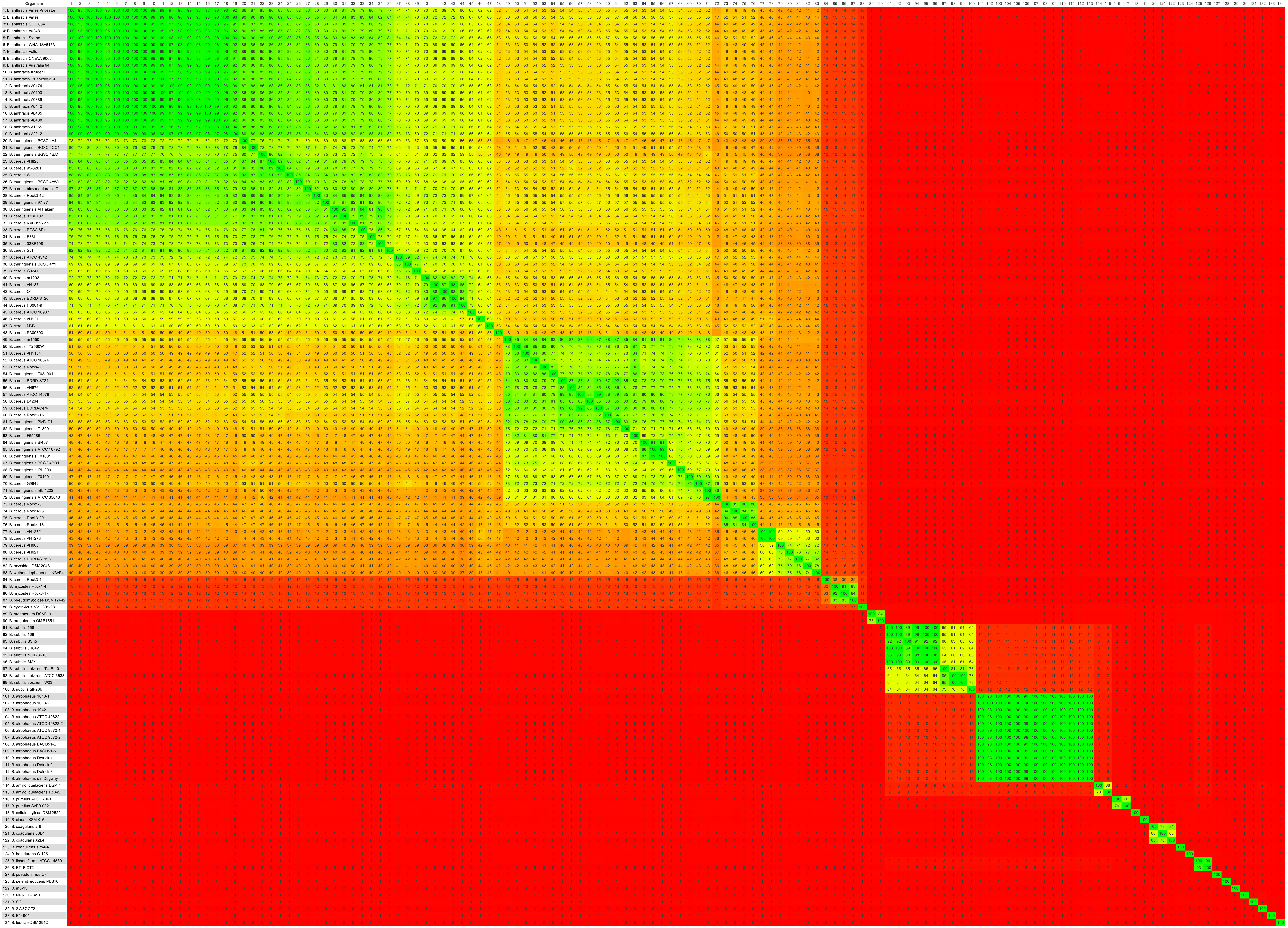
Organism
1: B. anthracis Ames Ancestor
2: B. anthracis Ames
3: B. anthracis CDC 684
4: B. anthracis A0248
5: B. anthracis Sterne
6: B. anthracis WNA USA6153
7: B. anthracis Vollum
8: B. anthracis CNEVA-9066
9: B. anthracis Australia 94
10: B. anthracis Kruger B
11: B. anthracis Tsiankovskii-I
12: B. anthracis A0174
13: B. anthracis A0193
14: B. anthracis A0389
15: B. anthracis A0442
16: B. anthracis A0465
17: B. anthracis A0488
18: B. anthracis A1055
19: B. anthracis A2012
20: B. thuringiensis BGSC 4AJ1
21: B. thuringiensis BGSC 4CC1
22: B. thuringiensis BGSC 4BA1
23: B. cereus AH820
24: B. cereus 95-8201
25: B. cereus W
26: B. thuringiensis BGSC 4AW1
27: B. cereus biovar anthracis CI
28: B. cereus Rock3-42
29: B. thuringiensis 97-27
30: B. thuringiensis Al Hakam
31: B. cereus 03BB102
32: B. cereus NVH0597-99
33: B. cereus BGSC 6E1
34: B. cereus E33L
35: B. cereus 03BB108
36: B. cereus SJ1
37: B. cereus ATCC 4342
38: B. thuringiensis BGSC 4Y1
39: B. cereus G9241
40: B. cereus m1293
41: B. cereus AH187
42: B. cereus Q1
43: B. cereus BDRD-ST26
44: B. cereus H3081-97
45: B. cereus ATCC 10987
46: B. cereus AH1271
47: B. cereus MM3
48: B. cereus R309803
49: B. cereus m1550
50: B. cereus 172560W
51: B. cereus AH1134
52: B. cereus ATCC 10876
53: B. cereus Rock4-2
54: B. thuringiensis T03a001
55: B. cereus BDRD-ST24
56: B. cereus AH676
57: B. cereus ATCC 14579
58: B. cereus B4264
59: B. cereus BDRD-Cer4
60: B. cereus Rock1-15
61: B. thuringiensis BMB171
62: B. thuringiensis T13001
63: B. cereus F65185
64: B. thuringiensis Bt407
65: B. thuringiensis ATCC 10792
66: B. thuringiensis T01001
67: B. thuringiensis BGSC 4BD1
68: B. thuringiensis IBL 200
69: B. thuringiensis T04001
70: B. cereus G9842
71: B. thuringiensis IBL 4222
72: B. thuringiensis ATCC 35646
73: B. cereus Rock1-3
74: B. cereus Rock3-28
75: B. cereus Rock3-29
76: B. cereus Rock4-18
77: B. cereus AH1272
78: B. cereus AH1273
79: B. cereus AH603
80: B. cereus AH621
81: B. cereus BDRD-ST196
82: B. mycoides DSM 2048
83: B. weihenstephanensis KBAB4
84: B. cereus Rock3-44
85: B. mycoides Rock1-4
86: B. mycoides Rock3-17
87: B. pseudomycoides DSM 12442
88: B. cytotoxicus NVH 391-98
89: B. megaterium DSM319
90: B. megaterium QM B1551
91: B. subtilis 168
92: B. subtilis 168
93: B. subtilis BSn5
94: B. subtilis JH642
95: B. subtilis NCIB 3610
96: B. subtilis SMY
97: B. subtilis spizizenii TU-B-10
98: B. subtilis spizizenii ATCC 6633
99: B. subtilis spizizenii W23
100: B. subtilis gtP20b
101: B. atrophaeus 1013-1
102: B. atrophaeus 1013-2
103: B. atrophaeus 1942
104: B. atrophaeus ATCC 49822-1
105: B. atrophaeus ATCC 49822-2
106: B. atrophaeus ATCC 9372-1
107: B. atrophaeus ATCC 9372-2
108: B. atrophaeus BACI051-E
109: B. atrophaeus BACI051-N
110: B. atrophaeus Detrick-1
111: B. atrophaeus Detrick-2
112: B. atrophaeus Detrick-3
113: B. atrophaeus str. Dugway
114: B. amyloliquefaciens DSM 7
115: B. amyloliquefaciens FZB42
116: B. pumilus ATCC 7061
117: B. pumilus SAFR 032
118: B. cellulosilyticus DSM 2522
119: B. clausii KSM K16
120: B. coagulans 2-6
121: B. coagulans 36D1
122: B. coagulans XZL4
123: B. coahuilensis m4-4
124: B. halodurans C-125
125: B. licheniformis ATCC 14580
126: B. BT1B CT2
127: B. pseudofirmus OF4
128: B. selenitireducens MLS10
129: B. m3-13
130: B. NRRL B-14911
131: B. SG-1
132: B. 2 A57 CT2
133: B. B14905
134: B. tusciae DSM 2912
