# Supplementary material for: Gegenees: Fragmented Alignment of Multiple Genomes for Determining Phylogenomic Distances and Genetic Signatures Unique for Specified Target Groups
Source: PLoS One. 2012 Jun 18;7(6):e39107. doi: 10.1371/journal.pone.0039107 (PMC3377601; doi:10.1371/journal.pone.0039107)
Supplement: Text S2 — Comparison between Gegenees, Mauve and Mugsy. Text describing an analysis example with Gegenees, Mauve and Mugsy. The highly plastic genomes of the available Helicobacter pylori strains were used to show how fragmented and anchored alignments performs and complements each other when used with highly rearranged sequences. (PDF) [file pone.0039107.s018.pdf]

Supplemental Text S2.

## Comparison between Gegenees, Mauve and Mugsy

To compare the performance of Gegenees, Mauve and Mugsy on challenging datasets, the available genome sequences from the species *Helicobacter pylori,* which has been shown to have a highly plastic genome [1,2], were used in an analysis example. The recombinations can clearly be seen in Mauve (e.g. Figure S2:1B). In contrast, Gegenees signatures are only based on differences in sequence similarity and are not affected by differences in genome location and synteny. Eighteen *H. pylori*-genomes (Table S2:1) were chosen as a dataset for the comparison of Gegenees with Progressive Mauve 2.3.1 [3] and Mugsy 1.2.3 [4]. The aim of the analysis was to compare the *H. pylori* Gambia94_24 genome with a set of other *H. pylori* genomes to identify unique genomic features of the Gambia94_24 strain.

Gegenees completed the alignment (500/500 settings) of the 18 genomes in 1 minute and 37 seconds, Progressive Mauve finished the same analysis on the same computer in 2 hours 27 minutes and Mugsy used 60 minutes. The output file produced by Mugsy was analyzed in Gmaj [5].

According to the phylogenomic overview in Gegenees, the Gambia94_24 genome is most closely related to *H. pylori* strain 2017. The close relation between these two genomes was also visible in Mauve where no inversions or rearrangements could be seen (Fig S2:1A). An example of a genome more distantly related to Gambia94_24 is the B8-genome (Fig S2:1B).

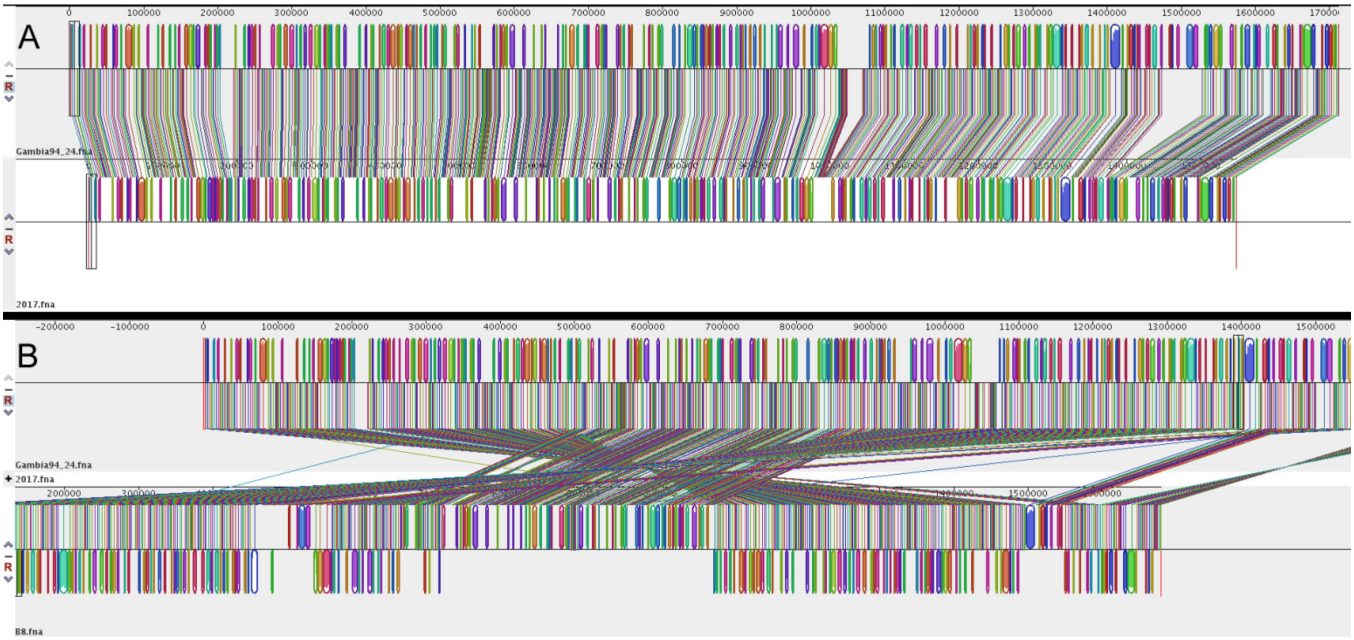


**Fgure S2:1**. Screenshots from the Mauve alignment of 18 *H. pylori* genomes. **A.** strain Gambia94_24 versus strain 2017. **B.** strain Gambia94_24 versus strain B8

When using Gambia94_24 as reference and the other 17 genomes as background in Gegenees, 30 fragments (each 500 bp) were identified with a biomarker score above 0.8. The majority of these were located in the region ~204,000 – 216,000 (Figure S2:2B). Some of the annotations in this region were phage-related indicating it represented a prophage. This region was also seen in Mauve as an unaligned area but the graphical overview of all 18 genomes were quite complex to interpret. The Mugsy-alignment viewed in Gmaj, on the other hand, gave a more easily interpretable overview from a signature identification perspective, although draft genomes gave multiple rows making them difficult to overview. In this analysis, the ~204,000 – 216,000 area was clearly shown as unaligned in the other genomes (marked with red arrow in Figure S2:2A). Two other areas were visible that had low representation in other genomes (marked with black arrow in Figure S2:2A). These semi-unique areas can be identified in Gegenees as well when using low stringency biomarker scores (Figure S2:2C).

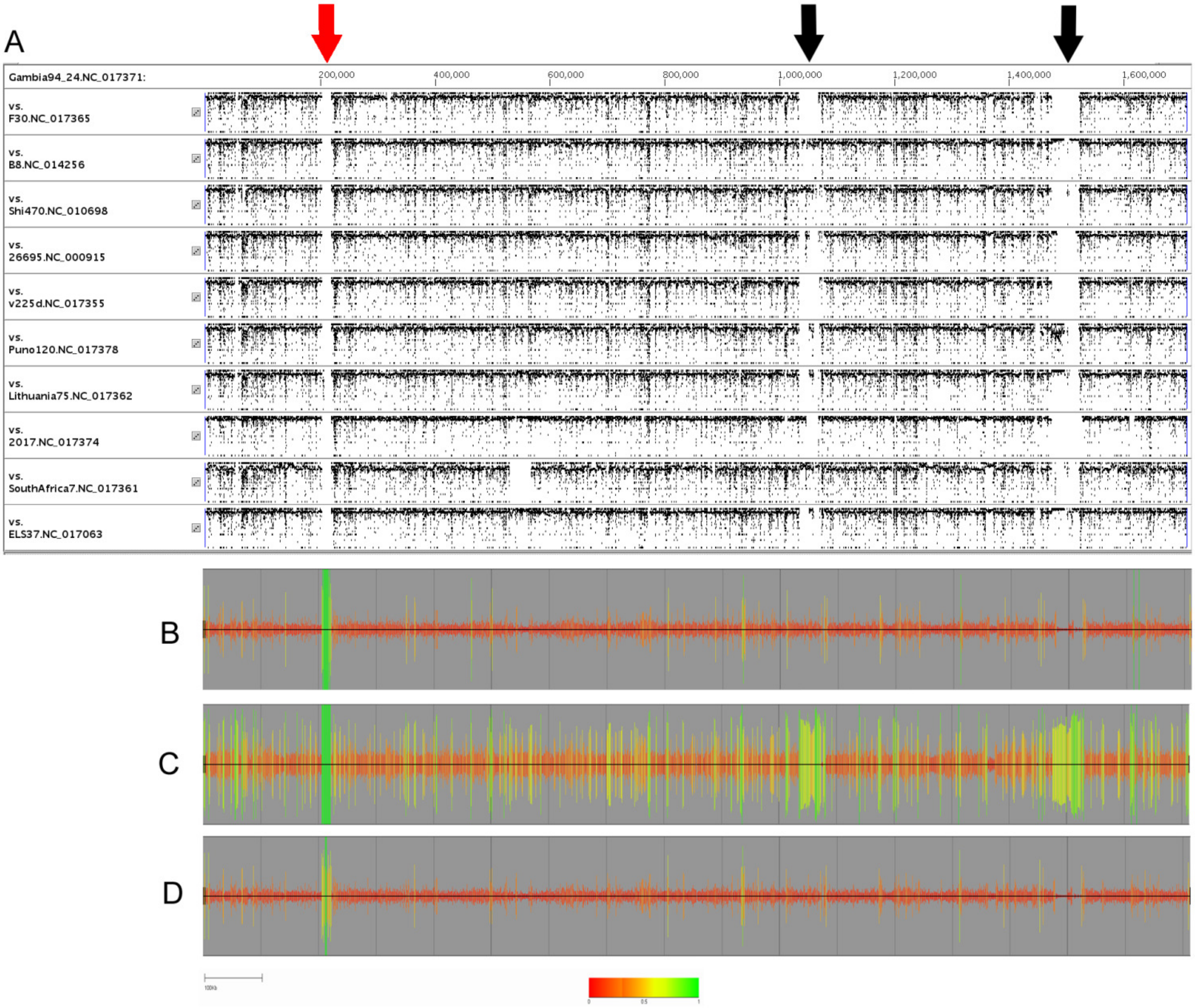


**Figure S2:2 A**. Screenshot from a Mugsy alignment of 18 *H. pylori* genomes viewed in Gmaj. **B.** Gegenees signature from an alignment of 18 *H. pylori* genomes. **C.** Same alignment as B but using low stringency biomarker scores. **D.** Same analysis as B but using 48 *H. pylori* genomes.

In conclusion, to rapidly see if there are any major unique genomic areas in a sequenced genome, both Mugsy and Gegenees were suitable. However, when using Mugsy, we could not include as many genomes as with Gegenees and the draft genomes were more difficult to analyze. Mauve gave informative graphs but they were complex to interpret in terms of signatures. Gegenees completed the alignment in less than 1/30 of the time Mugsy needed. Gegenees can also efficiently identify small signatures. In Mauve and Mugsy, only relatively large regions with signature values could be identified within the graphical views. Although this study was performed using 18 genomes, there were in fact 47 *H. pylori*-genomes available at the time and Gegenees aligned all of them in only 8 minutes which was 13 % of the time Mugsy needed for only 18 genomes. As seen in Figure S2:2D, the phage-region of the Gambia94_24 genome still comes out as unique when using all 46 *H. pylori*-genomes as background although its size is somewhat restricted. In summary, Gegenees signature analysis is fast and can handle many genomes but it can also be a good complement to use anchor-based alignments during a signature analysis.

**Table S2:1** The 18 *H. pylori* genomes used in this analysis

| Strain name | Complete/Draft | No of Subsequences | NCBI Accession number or WGS project code |
|---|---|---|---|
| Helicobacter_pylori_Puno120 | Complete | 2 | NC_017377, NC_017378 |
| Helicobacter_pylori_Gambia94_24 | Complete | 2 | NC_017364, NC_017371 |
| Helicobacter_pylori_26695 | Complete | 1 | NC_000915 |
| Helicobacter_pylori_F30 | Complete | 2 | NC_017365, NC_017369 |
| Helicobacter_pylori_2017 | Complete | 1 | NC_017374 |
| Helicobacter_pylori_NQ4060 | Draft | 59 | CADK |
| Helicobacter_pylori_HPKX_438_AG0C1 | Draft | 2602 | ABJO |
| Helicobacter_pylori_Lithuania75 | Complete | 2 | NC_017363, NC_017362 |
| Helicobacter_pylori_Shi470 | Complete | 1 | NC_010698 |
| Helicobacter_pylori_SouthAfrica7 | Complete | 2 | NC_017361, NC_017373 |
| Helicobacter_pylori_B8 | Complete | 2 | NC_014257, NC_014256 |
| Helicobacter_pylori_NQ4191 | Draft | 43 | CADN |
| Helicobacter_pylori_NQ1701 | Draft | 78 | CADH |
| Helicobacter_pylori_8A3 | Draft | 44 | CADD |
| Helicobacter_pylori_NQ367 | Draft | 90 | CADL |
| Helicobacter_pylori_NQ315 | Draft | 57 | CADE |
| Helicobacter_pylori_v225d | Complete | 2 | NC_017355, NC_017383 |
| Helicobacter_pylori_ELS37 | Complete | 2 | NC_017063, NC_017064 |

## References


1. Gressmann H, Linz B, Ghai R, Pleissner KP, Schlapbach R, et al. (2005) Gain and loss of multiple genes during the evolution of Helicobacter pylori. PLoS genetics 1: e43.
2. Suerbaum S, Smith JM, Bapumia K, Morelli G, Smith NH, et al. (1998) Free recombination within Helicobacter pylori. Proceedings of the National Academy of Sciences of the United States of America 95: 12619-12624.
3. Darling AE, Mau B, Perna NT (2010) progressiveMauve: multiple genome alignment with gene gain, loss and rearrangement. PLoS One 5: e11147.
4. Angiuoli SV, Salzberg SL (2011) Mugsy: fast multiple alignment of closely related whole genomes. Bioinformatics 27: 334-342.
5. Blanchette M, Kent WJ, Riemer C, Elnitski L, Smit AF, et al. (2004) Aligning multiple genomic sequences with the threaded blockset aligner. Genome research 14: 708-715.
